# Assessment of Cell-Type-Specific Excitatory Synaptic Strength in the Dorsolateral Striatum of Goal-Directed and Habitual Cocaine-Seeking Behavior

**DOI:** 10.1523/ENEURO.0392-25.2026

**Published:** 2026-05-22

**Authors:** Kaliana M. Veros, Maureen M. Timm, Peter J. West, Karen S. Wilcox, Kristen A. Keefe

**Affiliations:** ^1^Department of Pharmacology & Toxicology, University of Utah, Salt Lake City, Utah 84112; ^2^Interdepartmental Program in Neuroscience, University of Utah, Salt Lake City, Utah 84112; ^3^Anticonvulsant Drug Development Program, Department of Pharmacology & Toxicology, University of Utah, Salt Lake City, Utah 84112

## Abstract

With repeated exposure to addictive drugs, there is a shift from drug abuse to drug addiction that is mediated by the transition from goal-directed to habitual control. It is well known that the development of habitual control over behavior relies upon cell-type-specific synaptic changes in both D1 and D2 medium spiny neurons (MSNs) in dorsal striatum. Specifically, habitual behavior is mediated by increased synaptic strength in D1 and D2 MSNs in dorsolateral striatum (DLS), suggesting similar cell-type-specific synaptic changes may underlie the development of habitual cocaine-seeking behavior. However, cell-type-specific synaptic changes have not been evaluated in DLS in this context. Therefore, we trained male rats to self-administer cocaine in a self-administration paradigm that allows for differentiation of goal-directed versus habitual cocaine-seeking behavior. Moreover, we used a viral vector under a D2-specific promoter to fluorescently label D2 MSNs with eYFP in DLS. Evoked excitatory postsynaptic currents (EPSCs) were used to determine AMPA:NMDA receptor ratio and the rectification index. Surprisingly, we did not observe any significant differences in these measures in DLS of cocaine-seeking rats, regardless of whether cocaine-seeking was under habitual control. Interestingly, preliminary observations revealed significant changes in the paired-pulse ratio (PPR), suggesting that presynaptic mechanisms may be involved in the development of habitual control over cocaine-seeking. Overall, however, these results suggest there are no changes in postsynaptic strength of D2 MSNs in the DLS of rats with an extended history of cocaine self-administration and regardless of whether the cocaine-seeking is under goal-directed or habitual control.

## Significance Statement

The study of drug abuse and drug addiction represents a critical area of research with significant public health implications. Importantly, the underlying neurobiology of the transition between drug abuse and drug addiction is not well understood, and insights to this transition may aid in the development of novel treatment options. Behaviorally, the shift from goal-directed to habitual control is thought to underlie this transition. Much is known about the neurobiology of goal-directed and habitual behavior; however, the transition in the context of drug-seeking is not well defined. We observed no significant differences in measures of synaptic strength, suggesting such postsynaptic neuroplasticity in the dorsolateral striatum is not involved in this transition.

## Introduction

Drug addiction is a chronic relapsing disorder wherein drug use behavior becomes inflexible and persistent even in the face of adverse consequences (Vanderschuren and Everitt, 2004; Belin et al., 2013). Initially, drugs are obtained and consumed to intentionally experience a reinforcing outcome, and drug-seeking behaviors are thereby outcome motivated and flexible (Vanderschuren and Everitt, 2004; Ersche et al., 2016). As drug use continues, the behaviors involved in drug consumption become automatic in response to drug-associated stimuli (Everitt and Robbins, 2005; Ersche et al., 2016). As such, drug-related behavior is considered to undergo a shift from goal-directed to habitual performance (Pierce and Vanderschuren, 2010; Ersche et al., 2016). Therefore, understanding behavioral and neurobiological processes involved in the progression of drug-related behavior from goal-directed to habitual is critical.

Goal-directed and habitual instrumental behaviors are known to be mediated by distinct subregions within dorsal striatum—the dorsomedial and dorsolateral striatum (DMS and DLS), respectively (Packard and Knowlton, 2002; Yin et al., 2004, 2006, 2009; Dolan and Dayan, 2013; Lipton et al., 2019). Importantly, the development of habitual control over appetitive instrumental behavior is associated with changes in glutamatergic synapses of DLS (Yin et al., 2009; Lipton et al., 2019; Malvaez, 2020). Early studies demonstrated increases in excitatory synaptic strength with extended training, suggesting that potentiated glutamate signaling in DLS underlies habitual actions (Yin et al., 2009). Further, as medium spiny neurons (MSNs) in dorsal striatum are segregated into dopamine D1 receptor or D2 receptor-expressing MSNs (Calabresi et al., 2014), other studies examined cell-type-specific changes in DLS (Lovinger, 2010 ; Shan et al., 2014; O’Hare et al., 2016; Lipton et al., 2019). Interestingly, both D1 and D2 MSNs of the DLS exhibit synaptic strengthening after extended training, suggesting synaptic potentiation in both subpopulations may underlie habitual control over behavior (Yin et al., 2009; O’Hare et al., 2016). While these studies indicate cell-type-specific changes in DLS in relation to habitual control over appetitive instrumental behavior, little is known about cell-type-specific changes in MSNs of DLS associated with habitual drug-seeking behavior.

In cocaine self-administration paradigms, inactivation of DLS reverts habitual cocaine-seeking behavior to goal-directed, suggesting drug-related behaviors are similarly mediated by dorsal striatum (Zapata et al., 2010; Jones et al., 2022). Despite these observations, cell-type-specific mechanistic changes within DLS underlying habitual drug-related behaviors remain poorly defined. Mechanistic changes have been well studied in the nucleus accumbens (NAc) in the context of cocaine self-administration (Kalivas, 2009). Most notably, persistent cocaine craving is associated with insertion of inwardly rectifying Ca^2+^-permeable AMPA (CP-AMPA) receptors in the NAc, enhancing MSN excitability and strengthening synapses (Conrad et al., 2008; Wolf, 2016). Moreover, D1 MSNs exhibit CP-AMPA receptor insertion following 10 d of cocaine self-administration (Terrier et al., 2016; Zinsmaier et al., 2022). However, given the importance of DLS in habitual control over behavior, cell-type-specific changes in the context of habitual cocaine-seeking represent a critical area of study in understanding synaptic changes underlying transition from drug abuse to drug addiction.

Therefore, the objective of the present study was to determine if there are changes in glutamatergic synaptic transmission in DLS of rats characterized as goal-directed or habitual in their cocaine-seeking behavior. Rats were assessed for sensitivity to outcome devaluation following training on a chained cocaine self-administration paradigm to determine goal-directed or habitual control over cocaine-seeking (Zapata et al., 2010; Giangrasso et al., 2023). We then assessed synaptic strength in DLS via patch-clamp recordings of evoked excitatory postsynaptic currents (EPSCs) in cells expressing eYFP under a D2 dopamine receptor-specific promotor (i.e., D2 MSNs), as well as in unlabeled cells (i.e., D1 or D2 MSNs). In all cell types, we found that excitatory synaptic strength, assessed through AMPA:NMDA receptor ratio and rectification index (RI), was unchanged in rats characterized as goal-directed or habitual relative to yoked-saline controls. Interestingly, we did observe a statistically significant decrease in the paired-pulse ratio (PPR) of EPSCs in eYFP+ cells from habitual rats as compared with rats characterized as being goal-directed in their cocaine-seeking, as well as a significant increase in PPR of EPSCs in eYFP− MSNs relative to saline controls, indicating presynaptic release may be differentially altered onto MSN subpopulations. Overall, the present results suggest no change in excitatory synaptic input onto D2 or putative D1 MSNs in DLS of rats with a history of cocaine self-administration, regardless of whether the cocaine-seeking is under goal-directed or habitual control. Future studies thoroughly interrogating presynaptic release properties onto DLS MSNs are warranted, however, in light of the present preliminary findings of altered PPRs in rats with habitual control over their cocaine-seeking behavior.

## Materials and Methods

### Animals

Male Long–Evans rats (300–350 g) surgically prepared with right jugular vein catheters were obtained from Charles River Laboratories. Upon arrival, catheter patency was verified and maintained by daily infusions of heparin-dextrose catheter-locking solution (500 IU/50% dextrose; SAI Infusion) and daily infusions of prophylactic Baytril (10 mg/kg, i.v.; Norbrook). All rats underwent stereotaxic surgery 6–8 d after arrival and were allowed to recover for 5–7 d before the start of behavioral training. Rats were single-housed in standard housing conditions following surgery to prevent the rats from damaging the indwelling catheter and were randomly assigned to receive either cocaine training or to serve as yoked-saline controls. Three days before the start of cocaine self-administration training, rats were food-restricted to 25 g of standard chow per day, fed following training, with *ad libitum* access to water. Rats were maintained on this feeding schedule throughout experimental training. Experimental training was performed between ZT3 and ZT7. Animal care, surgeries, and experimental procedures followed the *Guide for the Care and Use of Laboratory Animals (8th Edition)* and were performed in accordance with the University of Utah's animal care committee's regulations.

### Stereotaxic surgery

Rats were anesthetized with isoflurane (2.0–2.5% induction; 1.5–2.0% maintenance) and placed in a stereotaxic apparatus (Stoelting). A small hole was drilled in the skull and a 28-gauge infusion cannula (P1 Technologies) was inserted into the DLS (in mm relative to bregma: AP +0.7, ML −3.6, DV −5.0; Yin et al., 2004; Giangrasso et al., 2023). In order to identify dopamine D2 receptor-expressing MSNs for patch-clamp studies, a total of 1 µl of AAV-8-D2SP-eYFP-WPRE (titer: 2.25 × 10^9^ viral particles; Stanford Gene Vector and Virus Core, GVVC ID: GVVC-AAV-114; Zalocusky et al., 2016) was infused into DLS over the course of 10 min, after which the cannula was left in place for an additional 5 min before removal to ensure maximal diffusion. Rats were given 10 mg/kg Baytril intravenously and 0.1 ml of heparin-dextrose catheter-locking solution (500 IU/50% dextrose; SAI Infusion) on the day of surgery. Rats also were given 5 mg/kg carprofen subcutaneously, for prophylactic pain management on the day of and for 3 d after surgery. Throughout the recovery period and cocaine self-administration training and testing, rats were given daily infusions of intravenous Baytril and heparin-dextrose catheter-locking solution following training. Rats were allowed to recover from stereotaxic surgery for 3–5 d before food restriction began and 5–7 d before the start of self-administration training.

### Cocaine self-administration paradigm

The cocaine self-administration paradigm, adapted from Zapata et al. (2010) and Giangrasso et al. (2023), was performed during the light cycle and conducted in eight standard operant chambers that were enclosed in sound- and light-attenuating cabinets (Coulbourn Instruments). One wall of the chamber had two retractable levers on the right and left sides, and the opposite wall was equipped with a 3 W, 24 V house light. Graphic State 4.0 software (Coulbourn Instruments) was used to control chamber equipment and experimental protocols, as well as to record the number of lever presses and session time. Rats were trained to press one lever (designated as the drug “taking” lever) for intravenous cocaine•HCl infusion (0.33 mg/50 µl infusion; calculated as the salt; NIDA Drug Supply Program) under a fixed ratio 1 (FR1) schedule. Each taking-lever response was accompanied by retraction of the taking lever, illumination of a cue light, and extinction of the house light for a 30 s time-out (TO) period. Rats were trained daily on the taking lever for 2 h or 40 infusions per session, whichever came first. A criterion of two consecutive sessions with >10 cocaine infusions per session was required before rats progressed to a chained seeking-taking schedule. In this phase of training, an additional, different lever, designated as the “seeking” lever, was introduced. The first press of the seeking lever, under a 2 s random interval (RI) schedule, resulted in retraction of the seeking lever and presentation of the taking lever. Responding on the taking lever was kept on the FR1 schedule of cocaine administration, followed by the 30 s TO period. Following the TO period, the seeking lever was reinserted, and the chained cycle began again. This seeking-taking chained schedule is denoted as: RI(2 s)/FR1:TO(30 s) or “Chain #1.” Rats underwent daily training on this schedule (3 h or 12 infusions, whichever came first) until they reached criterion of two consecutive sessions with 12 infusions/session. Rats then progressed through increasing chained schedules: Chain #2 RI(20 s)/FR1:TO(120 s) and Chain #3 RI(60 s)/ FR1:TO(300 s) using the same training criteria. They then advanced to Chain #4 RI(120 s)/FR1:TO(600 s), which continued for six sessions (12 infusions/session), at which point all rats had achieved stable responding (<20% variability in drug infusions). A subset of rats underwent training on Chain #4 for six additional days to promote habitual control over behavior. Yoked-saline controls received response-independent saline infusions identical to that of their paired experimental rat and lever-pressing had no scheduled consequences.

Upon completion of Chain #4, rats progressed to 13 d of outcome devaluation wherein only the taking lever was available, and no cocaine was delivered upon lever-pressing, thereby devaluing the drug-taking lever via outcome omission. After the last day of outcome devaluation, seeking behavior was assessed under these “devalued” conditions in a 5 min test session. In this seeking test, only the seeking lever was available and responding did not result in any consequences. Following the “devalued” seeking test, the taking lever was then revalued over two daily sessions identical to the initial FR1 taking-lever training sessions (2 h or 40 infusions, whichever came first). The following day, another 5 min seeking test was conducted under these “valued” conditions. Comparing the number of cocaine-seeking lever presses made under the “devalued” versus the “valued” test conditions gives insight into the sensitivity of drug-seeking to outcome devaluation, thereby enabling the classification of cocaine-seeking behavior as either under goal-directed or habitual control (Zapata et al., 2010; Giangrasso et al., 2023). A “cocaine-seeking score” was determined for each rat as the number of seeking responses made under the “devalued” test condition expressed as a percentage of seeking responses made under the “valued” test condition. Rats with cocaine-seeking scores ≤70% were classified as having “goal-directed” cocaine-seeking behavior, scores between 71 and 79% as “intermediate,” and scores ≥80% were classified as “habitual,” similar to that previously reported by others (Zapata et al., 2010; Giangrasso et al., 2023). All rats were killed within 1–4 d following completion of the final seeking test for electrophysiology recordings. We limited this time course to 1–4 d after the testing as work on incubation of cocaine craving showed no significant changes in drug-seeking over Days 1–4 (Grimm et al., 2001) and subsequent studies of incubation variably use 1–4 d as “early” withdrawal time points (Grimm et al., 2001; Lu et al., 2004). The 1–4 d period should, therefore, have mitigated potential confounding effects of withdrawal and incubation of craving on the dependent measures. Extended Data Figure 1-2 represents a diagram of the experimental study.

### Brain slice preparation

From the point of brain slice preparation onward through final data analysis, the experimenter was blinded to the treatment group and behavioral classification of the rats. Within 4 d after the final seeking test, rats were anesthetized with sodium pentobarbital (50 mg/kg, i.p.) and immediately decapitated. The brain was then divided into two hemispheres longitudinally. Coronal brain slices (350 µm) containing the DLS were collected in an oxygenated ice-cold NMDG-HEPES cutting solution (in mM: 92 NMDG, 2.5 KCl, 1.2 NaH_2_PO_4_, 30 NaHCO_3_, 20 HEPES, 25 glucose, 2 thiourea, 5 Na-ascorbate, 3 Na-pyruvate, 10 MgSO_4_, and 0.5 CaCl_2_). Slices were then transferred to a prewarmed (32–24°C) holding chamber containing NMDG-HEPES for 30 min as a protective recovery period. Stepwise, a Na+ spike-in procedure was carried out according to an optimal age-dependent schedule (Ting et al., 2014). After 30 min, slices were transferred to a holding chamber containing room-temperature oxygenated HEPES-aCSF holding solution (in mM: 92 NaCl, 2.5 KCl, 1.2 NaH_2_PO_4_, 30 NaHCO_3_, 20 HEPES, 25 glucose, 2 thiourea, 5 Na-ascorbate, 3 Na-pyruvate, 2 MgSO_4_, and 2 CaCl_2_). All solutions were bubbled with 95% O_2_/5% CO_2_ throughout the experiment, corrected to pH 7.30–7.35, and adjusted for an osmolarity between 295–300 mOsm.

### Electrophysiology

Whole-cell patch-clamp recordings of MSNs were obtained by recording in the voltage-clamp configuration through use of a MultiClamp 700B amplifier, a Digidata 1440A data acquisition board, and pClamp 10 software (Molecular Devices). Slices were visualized with a 40× water-immersion objective (NA 0.8; Carl Zeiss) using infrared differential interference contrast (IR-DIC) microscopy on an upright Axioskop 2 microscope (Carl Zeiss) prior to specific MSN identification. Cells displaying eYFP fluorescence (excitation 513 nm, emission 527 nm) deep in the tissue were targeted as eYFP+ cells for electrophysiology with patch pipettes containing a CsMeSO_3_ intracellular solution (in mM: 120 CsMeSO_3_, 15 CsCl, 8 NaCl, 10 HEPES, 0.2 EGTA, 10 TEA, 5 QX-314, 0.1 spermine, 4 MgATP, and 0.3 NaGTP; pH 7.28–7.33; osmolarity 290–295 mOsm). Cells not displaying eYFP fluorescence were targeted as eYFP− cells for electrophysiology. Resting membrane potential was determined in the *I* *=* *0* mode, and only cells with hyperpolarized resting membrane potentials (less than −65 mV) were included in analyses. Membrane and access resistance were monitored, and only stable recordings were included in the study.

A bipolar nichrome/formvar stimulating electrode placed 100–200 µm from the patch pipette in DLS was used to deliver stimulation to evoke EPSCs. A maximum of two MSNs (one eYFP+, one eYFP−) were patched per slice. Signals were acquired at 10 kHz and filtered at 2 kHz. Brain slices were recorded in oxygenated aCSF at 30°C (in mM: 126 NaCl, 2.5 KCl, 1 NaH_2_PO_4_, 26 NaHCO_3_, 10.5 glucose, 1.3 MgSO_4_, 2 CaCl_2_) containing picrotoxin (50 µM). EPSCs at holding potentials ranging from −70 mV through +40 mV were evoked using a stimulation intensity that elicited response amplitudes from 100 to 300 pA at −70 mV (0.4–1 mA). Evoked EPSC current–voltage relationships were determined by normalizing peak amplitude at each holding potential to the first evoked response at −70 mV. RI was determined as the ratio of peak EPSC amplitudes recorded at −70 mV to those recorded at +40 mV and used as a measure of the insertion of CP-AMPA receptors into the neurons. AMPA:NMDA receptor ratio was determined as the ratio of peak EPSC amplitudes recorded at −70 mV to peak amplitude 50–60 ms after stimulation at +40 mV (Shan et al., 2014). PPRs were determined as the ratio of peak amplitude of the second pulse to that of the first at −70 mV with an interstimulus interval (ISI) of 50 ms. All electrophysiological analyses were performed on baseline-corrected traces.

### RNAscope/immunohistochemistry

For verification of viral eYFP expression in D2 MSNs, a combination of RNAscope and immunohistochemistry (IHC) was used. Rats were anesthetized with sodium pentobarbital (50 mg/kg, i.p.), immediately decapitated, and the brain was flash frozen in 2-methylbutane (MilliporeSigma) and stored at −80°C until use. Brains were cryosectioned at 14 µm and thaw mounted on Superfrost Plus slides (VWR International). Coronal sections containing the dorsal striatum were processed using RNAscope (Advanced Cell Diagnostics a Bio-Techne Brand) following the manufacturer's protocol with probes against preproenkephalin (Penk1; RNAscope Probe-Rn-Penk, catalog #417431) as a marker for D2 MSNs or preprotachykinin (Tac1; RNAscope Probe- Rn-Tac1, catalog #450661) as a marker for D1 MSNs. Immediately following the RNAscope protocol, slides were incubated in blocking solution (0.1% Triton X-100, 10% normal goat serum in PBS) for 2 h. Slides were then incubated overnight in primary anti-GFP antibody (1:500 dilution, catalog #AB6556; Abcam; RRID:AB_305564) in blocking solution (0.1% Triton X-100, 1% normal goat serum in PBS). The primary antibody was visualized using an Alexa Fluor 488-conjugated secondary antibody (1:1,000 dilution, 0.1% Triton X-100, 1% normal goat serum in PBS, catalog #A11008; Invitrogen; RRID:AB_143165). Slides were coverslipped using ProLong Diamond with DAPI (catalog #P36971; Invitrogen). Areas of the DLS expressing GFP were first identified at 20× magnification. A confocal *z*-stack (10 µm; 1 µm *z*-steps) of the DLS containing GFP-expressing cells was then acquired at 63× magnification using a Zeiss LSM700 laser scanning confocal microscope (Carl Zeiss).

### Statistical analysis

All statistical analyses were performed in GraphPad Prism (GraphPad Software). For all analyses, alpha was set to 0.05, and power analyses were conducted with a desired power level of 0.8. D-Agostino and Pearson’s normality tests were performed on all data sets to determine if the data were normally distributed. If the data were normally distributed (*p* > 0.05), parametric tests were used, including one-way ANOVA and unpaired *t* tests. If the data were not normally distributed (*p* < 0.05), nonparametric versions of the aforementioned tests were used, including Kruskal–Wallis test, Mann–Whitney *U* test, and Spearman correlation. As noted above, all electrophysiology recordings and data analyses were conducted with the experimenter blinded to the treatment and behavioral classification of the subject. All statistical analyses are reported in the statistics table (Table 1).

**Table 1.** Statistics table

	Data structure	Test	Test statistic
a	Normal	One-way ANOVA	*F* = 0.1994
b	Non-normal	Kruskal–Wallis	*H* = 4.625
c	Non-normal	Mann–Whitney	*U* = 373
d	Normal	Unpaired *t* test	*t* = 0.0877
e	Normal	Unpaired *t* test	*t* = 0.942
f	Normal	Unpaired *t* test	*t* = 0.540
g	Non-normal	Mann–Whitney	*U* = 45
h	Non-normal	Kruskal–Wallis	*H* = 5.017
i	Normal	Unpaired *t* test	*t* = 1.6
j	Normal	One-way ANOVA	*F* = 0.511
k	Non-normal	Spearman	*r* = 0.3496
l	Non-normal	Spearman	*r* = −0.0035
m	Non-normal	Mann–Whitney	*U* = 31
n	Non-normal	Kruskal–Wallis	*H* = 6.151
o	Post hoc	Dunn's	*z* = 2.01; 0.17; 2.31
p	Non-normal	Mann–Whitney	*U* = 38
q	Non-normal	Kruskal–Wallis	*H* = 0.31
r	Non-normal	Spearman	*r* = 0.5242
s	Non-normal	Spearman	*r* = −0.068
t	Non-normal	Mann–Whitney	*U* = 5
u	Non-normal	Mann–Whitney	*U* = 19
v	Non-normal	Mann–Whitney	*U* = 37
w	Non-normal	Kruskal–Wallis	*H* = 6.705
x	Post hoc	Dunn's	*z* = 1.54; 1.16; 2.50
y	Non-normal	Mann–Whitney	*U* = 7
z	Non-normal	Kruskal–Wallis	*H* = 5.941
aa	Post hoc	Dunn's	*z* = 1.59; 2.41; 0.74
bb	Normal	Two-way ANOVA	Interaction: *F* = 0.038 Behavioral classification: *F* = 0.23 eYFP: *F* = 0.02
cc	Normal	Two-way ANOVA	Interaction: *F* = 0.27 Behavioral classification: *F* = 0.13 eYFP: *F* = 0.87
dd	Normal	Two-way ANOVA	Interaction: *F* = 0.009 Behavioral classification: *F* = 0.07 eYFP: *F* = 0.24

## Results

### Cocaine-seeking behavior

**Figure 1.**
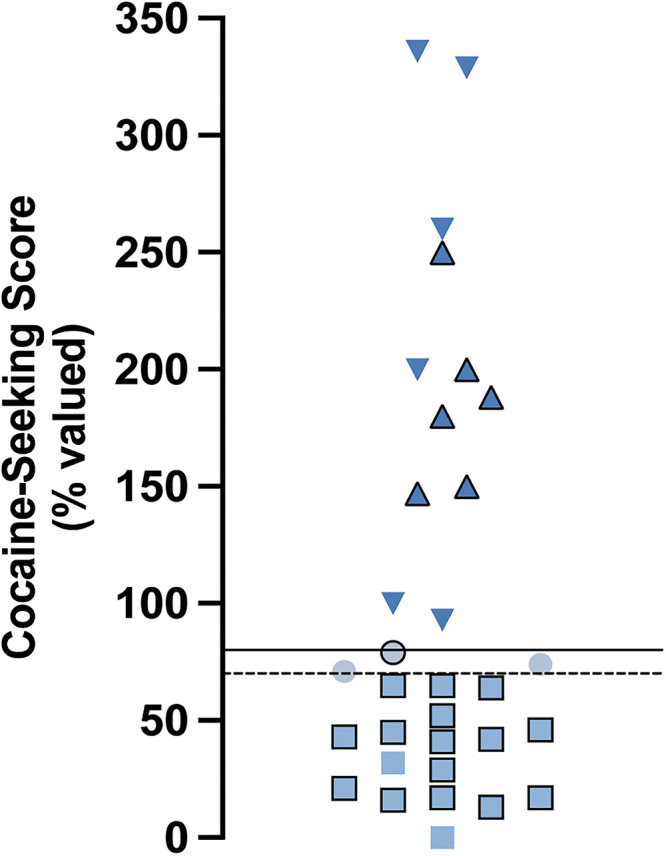
Cocaine-seeking scores of rats subjected to chained cocaine-seeking and cocaine-taking self-administration training. Distribution of cocaine-seeking scores (*n* = 32 rats; goal-directed: *n* = 17; intermediate: *n* = 3; habitual: *n* = 12). Cocaine-seeking scores (seeking responses under “devalued” conditions expressed as a percentage of responses under “valued” conditions) are classified as goal-directed (≤70%, squares), intermediate (71–79%, circles), and habitual (≥80%, triangles). Data points without borders or upside down represent rats trained an additional 6 d on Chain #4 (*n* = 10). Dashed line represents cocaine-seeking score of 70%, data points below this line are considered goal-directed. Solid line represents cocaine-seeking score of 80%, data points above this line are considered habitual. Taking-lever responses throughout self-administration training reported in Extended Data Figure 1-1. Full experimental timeline demonstrated in Extended Data Figure 1-2.

We used a chained cocaine self-administration paradigm, adapted from Zapata et al. (2010) and Giangrasso et al. (2023) to train male rats (*n* = 32 rats total) in cocaine-seeking and cocaine-taking behaviors (Extended Data Fig. 1-2). Through devaluation of drug-taking responses via outcome omission, the number of seeking-lever responses made under devalued and valued conditions were used to calculate the cocaine-seeking score and to classify cocaine-seeking behavior as either goal-directed (cocaine-seeking score ≤70%; *n* = 17), intermediate (cocaine-seeking score 71–79%; *n* = 3), or habitual (cocaine-seeking score ≥80%; *n* = 12; Fig. 1). All rats readily met criteria for cocaine-taking responses in initial training with a total average time to meet criteria on FR1 of 3.75 ± 0.42 d. The average number of days to meet criteria on the FR1 was similar between groups classified as goal-directed (4 ± 0.65 d; *n* = 17), intermediate (3.33 ± 1.33 d; *n* = 3), or habitual (3.5 ± 0.57 d; *n* = 12; one-way ANOVA: *F*_(2,29)_ = 0.199, *p* = 0.82^a^). All rats reduced responding during devaluation training, with no significant differences observed on the final day of devaluation training between goal-directed (13.1 ± 2.56 lever presses; *n* *=* 17), intermediate (29.3 ± 9.67 lever presses; *n* *=* 3), or habitual (49.5 ± 28.1 lever presses; *n* *=* 12) classifications (Extended Data Fig. 1-1; Kruskal–Wallis statistic = 4.625; *p* *=* 0.099^b^). Further, all rats readily reinstated cocaine-taking behavior in revaluation training sessions (22.8 ± 1.1 lever presses) to rates similar to those observed in initial FR1 training (20.8 ± 1.37 lever presses; Mann–Whitney *U* *=* 373; *p* *=* 0.061^c^; Extended Data Fig. 1-1). A subset (*n* = 10) of the animals referred to above were run on Chain #4 for an additional six days (i.e., Chain #4 for a total of 12 d) to promote habitual responding. Within this cohort, few remained goal-directed (*n* = 2) or intermediate (*n* = 2), whereas the majority were classified as habitual (*n* = 6) in their cocaine-seeking behavior. Following electrophysiology recordings and exclusions, the intermediate group was omitted from further analysis for lack of sufficient group size. Therefore, analysis of all electrophysiological data was performed on cells recorded in brain slices obtained from yoked-saline control rats (*n* = 7 rats) and cocaine self-administering rats classified as goal-directed (*n* = 11) and habitual (*n* = 10) in their cocaine-seeking behavior.

10.1523/ENEURO.0392-25.2026.f1-1 Figure 1-1 Taking-lever presses throughout cocaine self-administration training, devaluation (i.e., extinction), and revaluation sessions in rats ultimately classified as goal-directed (n = 11) or habitual (n = 10) in their cocaine-seeking behavior. All sessions were two-hours in length, with the exception that responding in the FR1 and Revaluation sessions was restricted to a maximum of 40 lever presses and, thus, could have been terminated at less than two hours. Data are represented as mean + SEM. Download Figure 1-1, TIF file.

**Figure 2.**
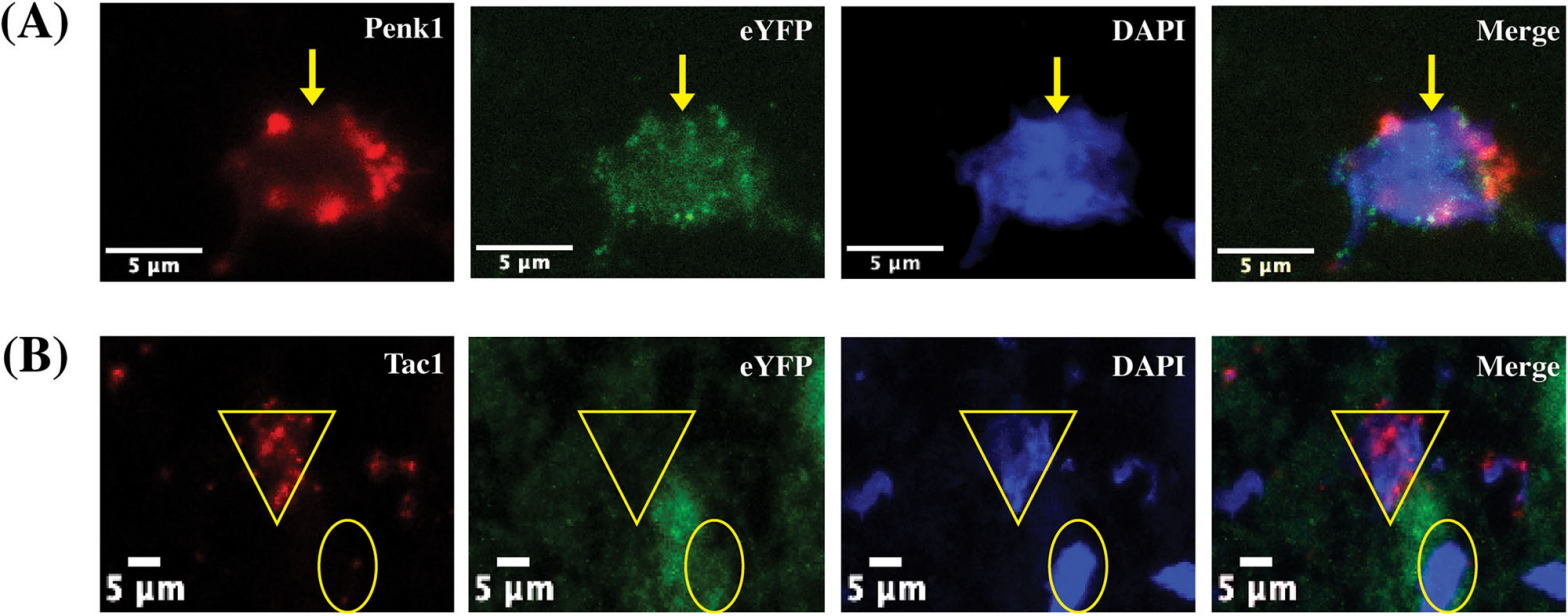
eYFP expression in identified D2 MSNs in the DLS. ***A***, A representative cell double-labeled for preproenkephalin (Penkl, red; D2 MSN, yellow arrow) and eYFP (green) with DAPI counterstain (blue) in the DLS. ***B***, A representative image showing eYFP expression (green; yellow circle) is not colocalized with preprotachykinin (Tac1, red; D1 MSN, yellow triangle) expressing cells.

10.1523/ENEURO.0392-25.2026.f1-2 Figure 1-2 Schematic of experimental paradigm. Rats arrived implanted with jugular vein catheters and underwent surgery within 3-5 days of arrival. Following recovery, rats then advanced to cocaine self-administration training. FR1 training duration was dependent upon meeting criteria of two consecutive sessions of >10 infusions. Within 4 days of the final testing session, rats were used in electrophysiology experiments. Download Figure 1-2, TIF file.

### Baseline electrophysiological characteristics of eYFP+ and eYFP− cells

To assess synaptic strength, we recorded stimulation-evoked EPSCs from both eYFP+ and eYFP− neurons in the DLS of acute brain slices obtained from yoked-saline controls and cocaine- experienced rats. We confirmed that eYFP expression was selective for D2 MSNs, rather than D1 MSNs, through RNAscope/IHC analysis of Penk1 and Tac1 mRNAs and eYFP protein expression. Importantly, Penk1 is a known marker of MSNs expressing D2 receptors, whereas Tac1 is known to be expressed in D1 MSNs throughout the striatum (Gerfen et al., 1990; Pollack and Wooten, 1992). RNAscope/IHC analysis revealed colocalization of Penk1/eYFP and lack of colocalization of Tac1/eYFP (Fig. 2), thereby suggesting that the viral eYFP expression is restricted to D2 MSNs.

In electrophysiological recordings, intrinsic properties were measured to ensure recordings were obtained from MSNs. MSNs were differentiated from other cell types (e.g., cholinergic interneurons) by a hyperpolarized resting membrane potential and low input resistance (Cepeda et al., 2008; Kreitzer and Malenka, 2008). Importantly, baseline characteristics (Table 2) were not different between voltage-clamped eYFP+ (*n* = 28 cells; 9 saline, 11 goal-directed, 8 habitual rats) and eYFP− (*n* = 21 cells; 6 saline, 7 goal-directed, 8 habitual rats) cells, including the following: resting membrane potential (unpaired t test; *p* *=* 0*.*93^d^), input resistance (unpaired t test; *p* *=* 0.35^e^), and evoked EPSC stimulation strength (unpaired t test; *p* *=* 0.59^f^). Further, a two-way ANOVA was performed to analyze the effect of behavioral classification on intrinsic properties of eYFP+ or eYFP− cells. There was no significant interaction between behavioral classification and eYFP status in resting membrane potential (*F*_(2,43)_ = 0.038; *p* = 0.96^bb^), input resistance (*F*_(2,43)_ = 0.27; *p* = 0.76^cc^), or evoked EPSC stimulation strength (*F*_(2,43)_ = 0.009; *p* = 0.99^dd^).

**Table 2.** Baseline electrophysiology characteristics of eYFP+ and eYFP− cells

Baseline electrophysiology characteristics	eYFP+	eYFP−
Resting membrane potential (mV)	−74.4 ± 0.7	−74.5 ± 0.8
Input resistance (MΩ)	168.6 ± 2.8	172.2 ± 2.5
Evoked EPSC stimulation strength (mA)	0.6 ± 0.04	0.6 ± 0.04

Together, these measures suggest that eYFP+ cells were D2 MSNs, that eYFP− cells were D1 MSNs or nontransfected D2 MSNs, and that eYFP expression does not influence electrophysiological characteristics of the recorded MSNs.

### Analyses of electrophysiological recordings reveal no changes in excitatory synaptic strength in MSNs expressing eYFP

**Figure 3.**
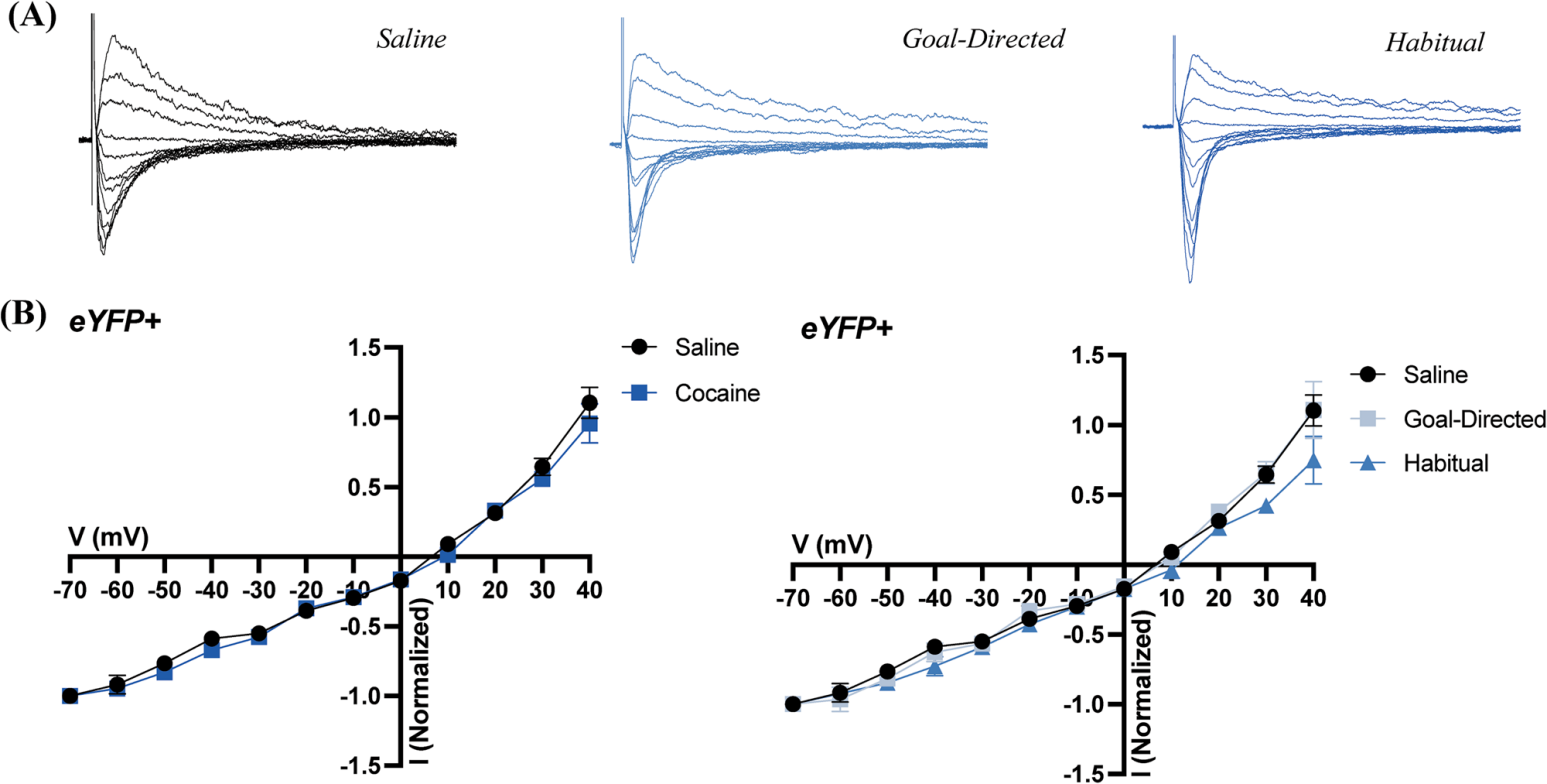
EPSCs in eYFP+ cells of the DLS obtained from acute brain slices of cocaine-experienced and yoked-saline control rats. ***A***, Representative normalized traces of evoked EPSCs in brain slices from control (left) and cocaine-experienced rats classified as goal-directed (middle) or habitual (right) in their cocaine-seeking behavior. ***B***, *I*/*V* relation of evoked EPSC responses between −70 and +40 mV in the DLS of eYFP+ cells between saline (*n* = 8 cells, 7 animals) and cocaine-experienced rats (left; *n* = 19 cells, 16 animals) classified as goal-directed (*n* = 11 cells, 10 animals) or habitual (*n* = 8 cells, 6 animals) in their drug-seeking behavior (right).

Analyses of EPSCs are common in the assessment of synaptic strength. Evoked EPSCs at a range of holding potentials provide insight to the relative distribution of AMPA and NMDA receptors, as well as the general current–voltage relationship of postsynaptic neurons. Therefore, we recorded evoked EPSCs in voltage-clamped MSNs in the DLS of acute brain slices obtained from yoked-saline controls and rats classified as goal-directed or habitual in their cocaine-seeking behavior. Evoked EPSCs were recorded at a range of voltages (−70 to +40 mV) to assess changes in various synaptic parameters. In D2 MSNs there were no apparent differences in the current–voltage relationship of evoked EPSCs between saline and cocaine-experienced rats when comparing normalized EPSCs at holding potentials between −70 and +40 mV (Fig. 3*B*). Likewise, no differences were apparent when recordings obtained from cocaine-experienced rats were broken down based on their behavioral classification as either goal-directed or habitual in their cocaine-seeking (Fig. 3*A*,*B*).

**Figure 4.**
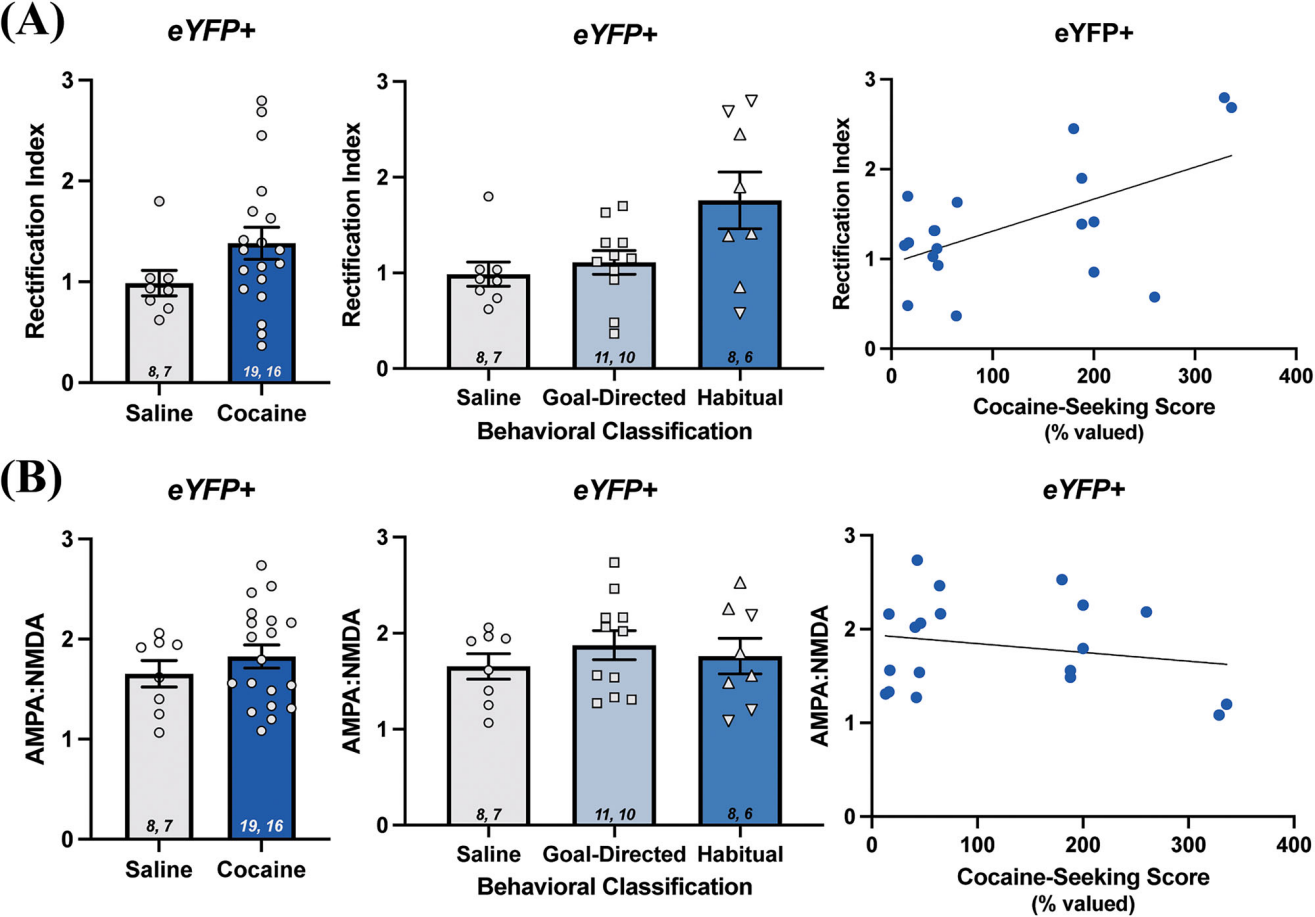
Measures of synaptic strength in eYFP+ cells of the DLS of rats with a history of cocaine self-administration. ***A***, Rectification index in brain slices from saline controls and rats with a history of cocaine self-administration (left; Mann–Whitney *U* test; *p* > 0.05) and in cocaine-experienced rats characterized as goal-directed or habitual in their cocaine-seeking behavior (middle; Kruskal–Wallis test; *p* > 0.05). Correlation between rectification index and cocaine-seeking score (right; Spearman *r* = 0.35; *p* > 0.05). ***B***, AMPA:NMDA receptor ratio in eYFP+ cells of saline controls and rats with a history of cocaine self-administration (left; unpaired *t* test; *p* > 0.05) and in goal-directed and habitual classifications (middle: one-way ANOVA; *p* > 0.05). Correlation between AMPA:NMDA receptor ratio and cocaine-seeking score (right; Spearman *r* = −0.004; *p* > 0.05). Data expressed as the mean + SEM. Sample sizes for each group denoted in each corresponding bar as: number of cells, number of animals. Upside down triangles represent data points from animals trained an additional six days on Chain #4. Correlation data fit with a linear regression line of best fit.

The insertion of inwardly rectifying CP-AMPA receptors has been demonstrated in the NAc following extended cocaine self-administration and withdrawal and is associated with incubation of cocaine craving (Wolf, 2016). Therefore, we hypothesized that insertion of CP-AMPA receptors in the DLS following cocaine exposure may similarly contribute to the development of habitual control over cocaine-seeking behavior. To assess whether there might be differences in the insertion of CP-AMPA receptors in the DLS of rats whose cocaine-seeking behavior was habitual, we examined the RI from evoked EPSCs. As CP-AMPA receptors are inwardly rectifying, an increase in the RI is interpreted as indicative of insertion of CP-AMPA receptors into the cell membrane. However, analyses of the RI did not reveal any differences in eYFP+ cells of the DLS from yoked-saline controls (0.99 ± 0.13; *n* = 8 cells) versus rats with a history of cocaine self-administration (1.38 ± 0.16; *n* = 19 cells; Mann–Whitney *U* = 45; *p* = 0.11^g^; Fig. 4*A*). Similarly, there were no differences in RI when the cocaine self-administering rats were grouped based on their behavioral classification as being goal-directed (1.11 ± 0.12; *n* = 11 cells) or habitual (1.76 ± 0.30; *n* = 8 cells) in relation to the yoked-saline controls (0.99 ± 0.13; *n* = 8 cells) or each other (Kruskal–Wallis statistic = 5.02; *p* = 0.08^h^; Fig. 4*A*). These findings suggest that AMPA receptor subunit composition and CP-AMPA receptor insertion are likely unchanged in D2 MSNs of the DLS of rats with a history of cocaine self-administration, regardless of whether their cocaine-seeking behavior is classified as goal-directed or habitual.

Increases in synaptic strength have been observed in the NAc following cocaine exposure, as well as in the DLS in the context of habitual instrumental behaviors. Therefore, we next assessed AMPA:NMDA receptor ratio as a measure of synaptic strength (Glasgow et al., 2019). AMPA receptor-mediated EPSCs were obtained at −70 mV, and NMDA receptor-mediated EPSCs were obtained 50–60 ms after the stimulus artifact when cells were held at +40 mV (Shan et al., 2014). AMPA:NMDA receptor ratios were not significantly different between yoked-saline controls (1.65 ± 0.13; *n* = 8 cells) and rats with a history of cocaine self-administration (1.83 ± 0.11; *n* = 19 cells; unpaired *t* test; *t* = 0.88; *p* = 0.39^i^; Fig. 4*B*). Further, AMPA:NMDA receptor ratios were not significantly different between the yoked-saline controls and the cocaine-experienced rats classified as goal-directed (1.88 ± 0.15; *n* = 11 cells) or habitual (1.76 ± 0.18; *n* = 8 cells) or when compared with each other (one-way ANOVA, *F*_(2,24)_ = 0.51; *p* = 0.61^j^; Fig. 4*B*). Taken together, the AMPA:NMDA receptor ratio measurements in addition to the RI and EPSC analyses indicate that synaptic strength is unaltered in D2-MSNs of the DLS in rats with a history of cocaine self-administration and regardless of whether the cocaine-seeking behavior is under goal-directed or habitual control.

As we observed a wide range of cocaine-seeking scores in our animals, we also performed a correlation analysis between cocaine-seeking scores and measures of both RI and AMPA:NMDA receptor ratios (Fig. 4). There was no significant correlation between cocaine-seeking scores and RI values in eYFP+ cells (Spearman *r* = 0.3496; *p* = 0.14^k^). Moreover, there was no significant correlation between cocaine-seeking scores and AMPA:NMDA receptor ratios (Spearman *r* = −0.0035; *p* = 0.99^l^).

### Electrophysiological recordings of MSNs not expressing eYFP reveal minimal changes in excitatory synaptic strength

**Figure 5.**
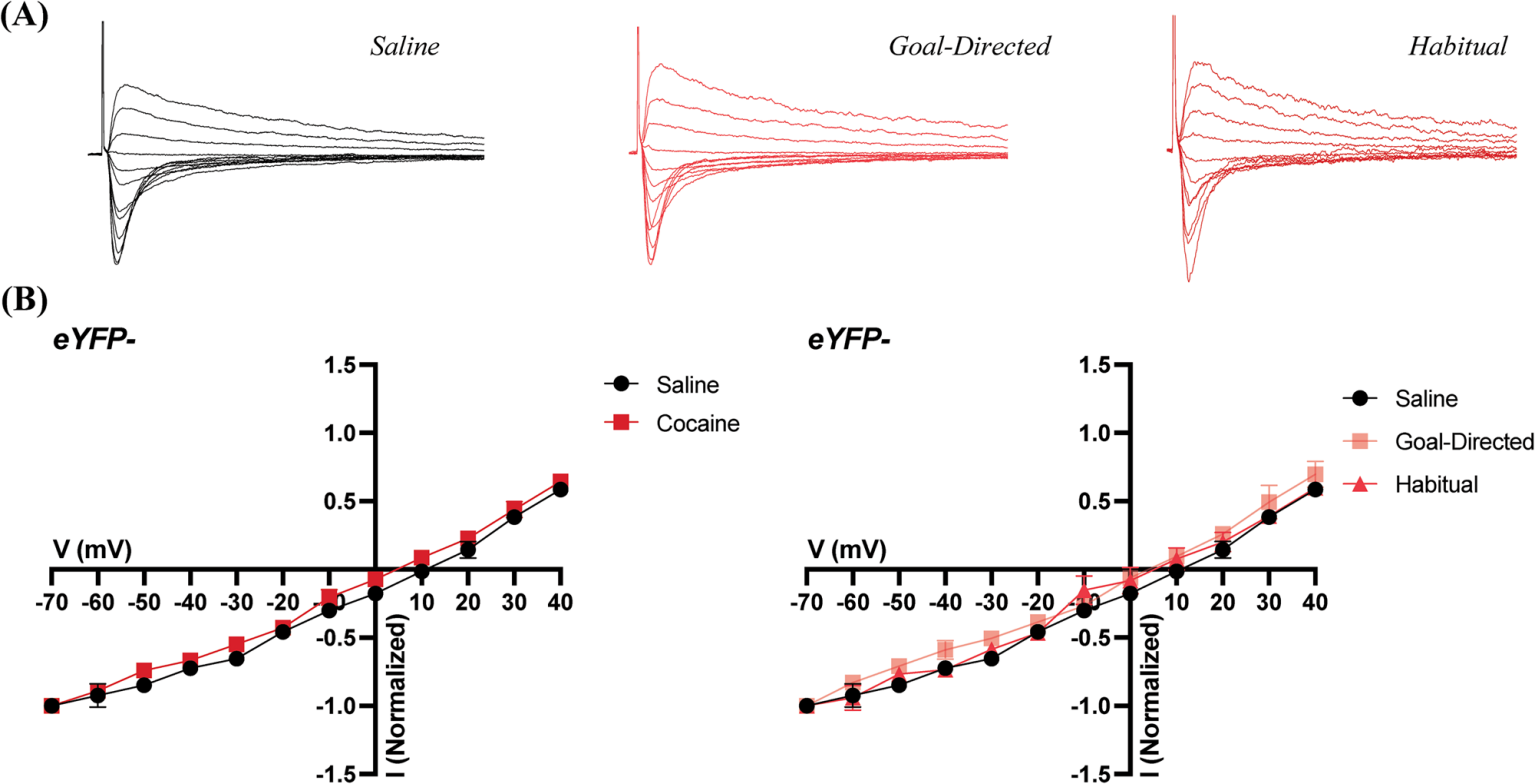
EPSCs in eYFP− cells of the DLS obtained from acute brain slices of cocaine-experienced and yoked-saline control rats. ***A***, Representative normalized traces of evoked EPSCs in brain slices from control (left) and cocaine-experienced rats classified as goal-directed (middle) or habitual (right) in their cocaine-seeking behavior. ***B***, *I*/*V* relation of evoked EPSC responses between −70 and +40 mV in the DLS of eYFP− cells between saline (*n* = 6 cells, 6 animals) and cocaine-experienced rats (left; *n* = 14 cells, 14 animals) classified as goal-directed (*n* = 6 cells, 6 animals) or habitual (*n* = 8 cells, 8 animals) in their drug-seeking behavior (right).

**Figure 6.**
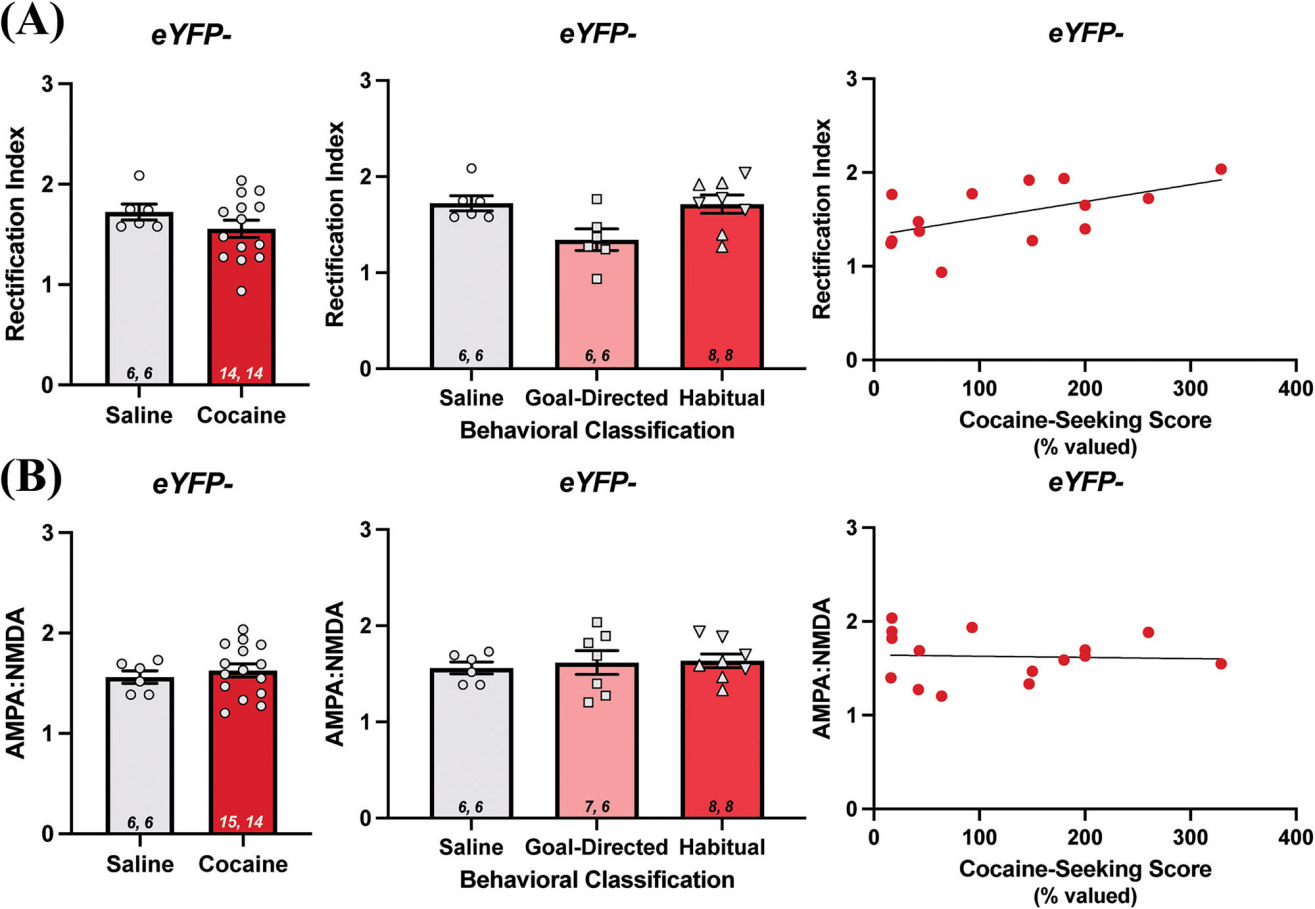
Measures of synaptic strength in eYFP− cells of the DLS of rats with a history of cocaine self-administration. ***A***, Rectification index in brain slices from saline controls and rats with a history of cocaine self-administration (left; Mann–Whitney *U* test; *p* > 0.05), and in cocaine-experienced rats characterized as goal-directed or habitual in their cocaine-seeking behavior (middle; Kruskal–Wallis test; *p* = 0.05; Dunn's post hoc multiple-comparisons adjusted *p* values; vs goal-directed: *p* > 0.05, vs habitual: *p* > 0.05, goal-directed vs habitual: *p* > 0.05). Correlation between rectification index and cocaine-seeking score (right; Spearman *r* = 0.52; *p* > 0.05). ***B***, AMPA:NMDA receptor ratio in eYFP− cells of saline controls and rats with a history of cocaine self-administration (left; Mann–Whitney *U* test; *p* > 0.05) and in goal-directed and habitual classifications (middle: Kruskal–Wallis; *p* > 0.05). Correlation between AMPA:NMDA receptor ratio and cocaine-seeking score (right; Spearman *r* = −0.07; *p* > 0.05). Data expressed as the mean + SEM. Sample sizes for each group denoted in each corresponding bar as: number of cells, number of animals. Upside down triangles represent data points from animals trained an additional six days on Chain #4. Correlation data fit with a linear regression line of best fit.

Within the same brain slices from which recordings from eYFP+ cells in the DLS were obtained, we also recorded from cells not expressing eYFP, which could be either D1-MSNs or nontransfected D2-MSNs. Analysis of the current–voltage relationships in eYFP− cells of yoked-saline controls and rats with a history of cocaine self-administration revealed no overall changes in EPSCs (Fig. 5). Specifically, as in the eYFP+ neurons, the current–voltage relationship between −70 and +40 mV did not display any apparent differences (Fig. 5*B*). We next assessed the RI, as described above. Again, there were no significant differences in RI between yoked-saline controls (1.72 ± 0.08; *n* = 6 cells) and rats with a history of cocaine self-administration (1.56 ± 0.09; *n* = 14 cells; Mann–Whitney *U* = 31; *p* = 0.40^m^; Fig. 6*A*). However, comparison of the RIs across the yoked-saline controls and cocaine self-administering rats broken down into goal-directed (1.34 ± 0.1; *n* = 6 cells) and habitual (1.71 ± 0.09; *n* = 8 cells) classifications did reveal an overall significant effect of classification (Kruskal–Wallis statistic = 6.1; *p* = 0.04^n^). Post hoc analysis, however, failed to identify significant differences between the yoked-saline controls and either behavioral classification of cocaine-experienced rats or between cells from rats classified as either goal-directed or habitual in their cocaine-seeking behavior (Dunn’s multiple-comparisons test adjusted *p* values; vs goal-directed: *p* = 0.09, vs habitual: *p* *>* *0.9*; goal-directed vs habitual: *p* = 0.06^o^; Fig. 6*A*). These findings therefore suggest that there are no AMPA receptor subunit changes or insertion of CP-AMPA receptors in eYFP− cells in the DLS of rats with a history of cocaine self-administration, regardless of whether the rats are goal-directed or habitual in their cocaine-seeking behavior.

Analysis of AMPA:NMDA receptor ratios in cells not expressing eYFP from saline (1.56 ± 0.06; *n* = 6 cells) and cocaine-experienced (1.63 ± 0.07; *n* = 15 cells) rats likewise revealed no significant differences in the ratios (Mann–Whitney *U* = 38; *p* = 0.62^p^; Fig. 6*B*). Similarly, there were no differences in AMPA:NMDA receptor ratios when the rats with the history of cocaine self-administration were categorized as goal-directed (1.62 ± 0.12; *n* = 7 cells) or habitual (1.64 ± 0.07; *n* = 8 cells) in their cocaine-seeking in relation to each other and the yoked-saline controls (Kruskal–Wallis statistic = 0.31; *p* = 0.86^q^; Fig. 6*B*). Together, the lack of differences in the RI and AMPA:NMDA receptor ratios between yoked-saline controls and cocaine-experienced rats categorized as goal-directed or habitual in their cocaine-seeking indicate that synaptic strength in eYFP− neurons of DLS is also unaffected by a history of cocaine self-administration, regardless of whether the cocaine-seeking behavior is characterized as goal-directed or habitual.

Similar to analyses performed in eYFP+ cells, we also performed correlation analyses between cocaine-seeking scores and measures of RI values and AMPA:NMDA receptor ratios in the eYFP− cell population (Fig. 6). We did not observe a significant correlation between cocaine-seeking scores and RI values (Spearman *r* = 0.5242; *p* = 0.0567^r^). Similarly, we did not observe a significant correlation between cocaine-seeking scores and AMPA:NMDA receptor ratio (Spearman *r* = −0.068; *p* = 0.81^s^).

Despite no significant differences observed in RI values and AMPA:NMDA receptor ratios between saline and cocaine-experienced rats, we did observe a significant difference in the RI values between eYFP+ and eYFP− neurons of the yoked-saline controls (compare Figs. 4*A*, 6*A*). RI values obtained from the eYFP− cells (1.72 ± 0.08) were significantly greater than those from eYFP+ cells (0.99 ± 0.13; Mann–Whitney *U* test, *p* < 0.05^t^). This observation suggests that the eYFP+ and eYFP− cells are distinct cellular populations (presumed D2 vs D1 MSNs), and eYFP− cells exhibit more pronounced inward rectification in comparison with eYFP+ cells. In contrast, AMPA:NMDA receptor ratio values did not differ between eYFP+ (1.65 ± 0.13) and eYFP− (1.56 ± 0.06^u^) cells from the saline control rats (compare Figs. 4*B*, 6*B*). Together, these findings suggest that D2 MSNs and presumed D1 MSNs display inherent differences in expression of CP-AMPA receptors but display no differences in synaptic strength under drug-naive conditions.

### Paired-pulse ratios reveal significant differences in both eYFP+ and eYFP− cells in the DLS of rats with a history of cocaine self-administration and classified as goal-directed or habitual in their cocaine-seeking behavior

**Figure 7.**
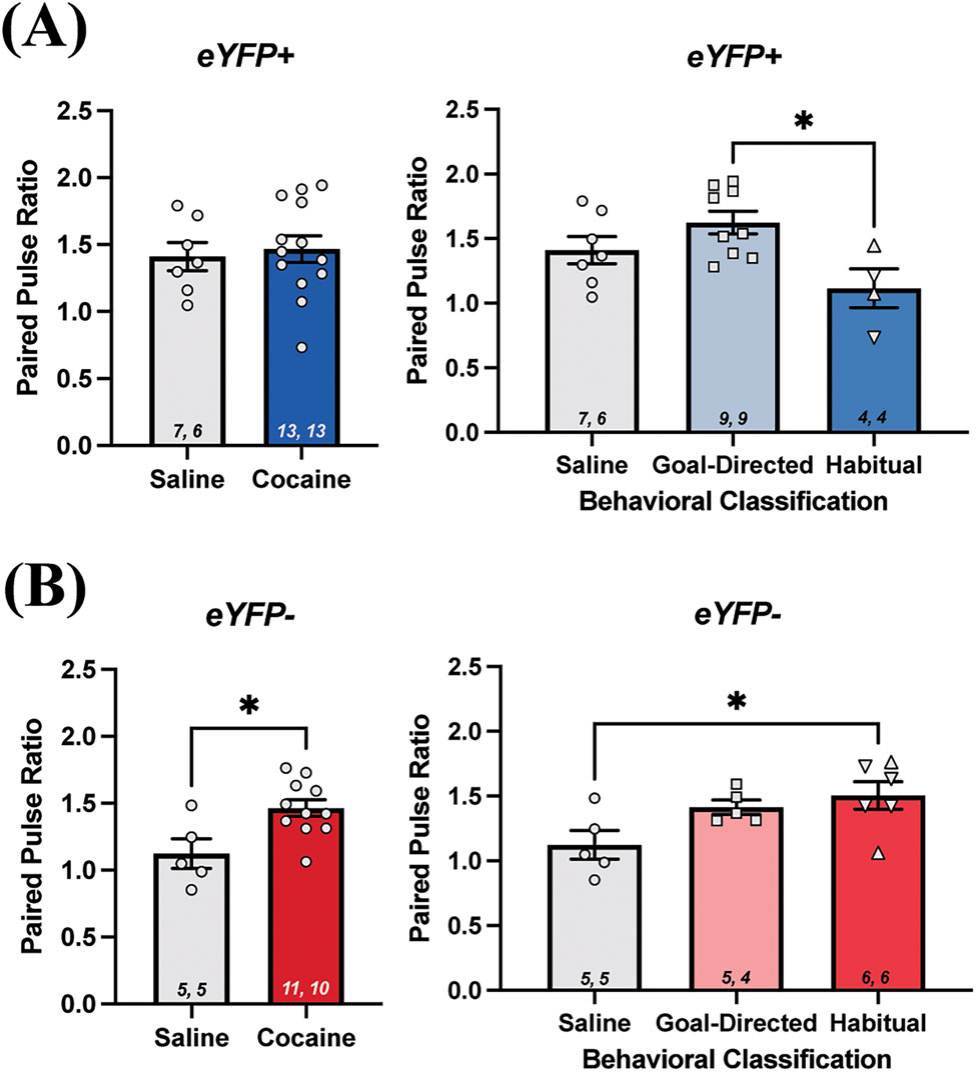
Paired-pulse ratio (PPR) in the DLS of yoked-saline control and cocaine-experienced rats classified as goal-directed or habitual in their cocaine-seeking behavior. ***A***, PPR values in brain slices from eYFP+ cells between control and cocaine-experienced rats (left; Mann–Whitney *U* test; *p* > 0.05), as well as between goal-directed and habitual behavioral classifications (right; Kruskal–Wallis test with Dunn's post hoc test, *p* < 0.05). ***B***, PPR values from eYFP− cells obtained from control and cocaine-experienced rats (left; Mann–Whitney *U* test; *p* < 0.05), and goal-directed or habitual classifications (right; Kruskal–Wallis test with Dunn's post hoc test, *p* < 0.05). Data expressed as mean + SEM. Sample sizes for each group denoted in each corresponding bar as: number of cells, number of animals. Upside down triangles represent data points from animals trained an additional sex days on Chain #4.

Within the same recording session, we obtained PPRs as a measure of presynaptic short-term synaptic plasticity. Measures of the PPR in D2 MSNs were not significantly different between yoked-saline controls (1.41 ± 0.10; *n* = 7 cells) and rats with a history of cocaine self-administration (1.47 ± 0.1; *n* = 13 cells; Mann–Whitney *U* = 37; *p* = 0.54^v^; Fig. 7*A*). In contrast, when PPR was assessed across rats classified as goal-directed (1.62 ± 0.09; *n* = 9 cells) or habitual (1.12 ± 0.15; *n* = 4 cells) in their cocaine-seeking behavior, the analysis revealed an overall significant effect (Kruskal–Wallis statistic = 6.7; *p* = 0.03^w^; Fig. 7*A*). The post hoc analysis did not reveal significant differences between goal-directed or habitual rats relative to the yoked-saline controls (Dunn’s multiple-comparisons test adjusted p values; goal-directed: *p* = 0.37, habitual: *p* = 0.74^x^; Fig. 7*A*). However, a significant difference was observed in the PPRs between the goal-directed and habitual groups, with the PPR of the habitual group being significantly lower than that in the goal-directed group (Dunn’s multiple-comparisons test adjusted *p* value = 0.04^x^; Fig. 7*A*), suggesting paired-pulse facilitation is weaker in D2 MSNs in the DLS of rats classified as habitual in their cocaine-seeking behavior than in those classified as goal-directed in their cocaine-seeking behavior. Decreased PPR is consistent with an increase in the probability of release of synaptic vesicles upon the first stimulation event from presynaptic afferent projections, reducing the number of vesicles available for release upon the next stimulation event, and thereby reducing the amplitude of the second evoked EPSC in D2 MSNs in the DLS of rats that are habitual in their cocaine-seeking behavior.

In contrast to eYFP+ neurons, PPRs from eYFP− neurons in DLS of rats with a history of cocaine self-administration (1.46 ± 0.06; *n* = 11 cells) were significantly greater than those in yoked-saline controls (1.12 ± 0.11; *n* = 5 cells; Mann–Whitney *U* = 7; *p* = 0.02^y^; Fig. 7*B*). Furthermore, analysis across yoked-saline controls and cocaine self-administering rats classified as goal-directed (1.4 ± 0.06; *n* = 5 cells) or habitual (1.5 ± 0.11; *n* = 6 cells) in their cocaine-seeking revealed an overall significant effect (Kruskal–Wallis statistic = 5.9; *p* = 0.04^z^; Fig. 7*B*). Post hoc analysis revealed that the PPRs of rats classified as habitual, but not goal-directed, in their cocaine-seeking behavior were significantly greater than those in eYFP− neurons from the yoked-saline controls (Dunn’s multiple-comparisons test adjusted *p* values; goal-directed: *p* = 0.33, habitual: *p* < 0.05^aa^; Fig. 7*B*). Together, the PPR values recorded from eYFP− neurons in the DLS suggest the possibility that a history of cocaine-seeking and cocaine-taking leading to habitual control over drug-seeking behavior is associated with a decrease in the initial probability of presynaptic glutamate release onto the eYFP− neurons.

## Discussion

The transition from goal-directed to habitual control over drug-related behavior is thought to play a critical role in the transition between recreational drug use and addiction. While much is known about cell-type-specific changes in synaptic strength in the DLS in the development of habitual behavior, specifically increasing synaptic strength of both D1 and D2 MSNs (Yin et al., 2009; O’Hare et al., 2016; Lipton et al., 2019), relatively little is known about the state of excitatory synaptic function in MSNs of the DLS in the context of habitual cocaine-seeking behavior. Therefore, the present study was designed to investigate changes in cell-type-specific synaptic strength induced by a history of cocaine self-administration and in relation to goal-directed versus habitual control over cocaine-seeking behavior. Overall, we found that synaptic strength, either through changes in the insertion of CP-AMPA receptors as reflected in changes in rectification index or in AMPA:NMDA receptor ratios, is unaffected in eYFP+ D2 MSNs and in other unlabeled MSNs by a history of cocaine self-administration under a seeking-taking paradigm and regardless of whether the cocaine-seeking is characterized as goal-directed or habitual. We did, however, observe preliminary evidence of short-term plasticity, as measured by PPRs, with there being a decrease in PPRs in eYFP+ neurons from rats whose cocaine-seeking was classified as habitual as compared with those classified as goal-directed and an increase in PPRs in eYFP− neurons from cocaine-experienced rats, specifically those classified as habitual in their cocaine-seeking behavior. Together, the present study represents the first assessment of cell-type-specific adaptations in excitatory synaptic transmission in MSNs of DLS in the context of habitual control over cocaine-seeking behavior and provides a foundation for future inquiries into the synaptic perturbations induced by repeated cocaine use.

The present study capitalized on a cocaine self-administration paradigm previously established and replicated in the literature by Zapata et al. (2010) and Giangrasso et al. (2023). This paradigm reliably produces rats whose cocaine-seeking behavior can be classified as goal-directed (cocaine-seeking score of ≤70%), intermediate (cocaine-seeking score between 71 and 79%) or habitual (cocaine-seeking score of ≥80%) based on sensitivity to devaluation of the cocaine-taking lever via outcome omission. Zapata et al. (2010) reported rats with goal-directed cocaine-seeking behavior transitioned to habitual control with extended training. Similarly, to increase the number of rats meeting criterion for being classified as habitual in their cocaine-seeking, a subset of our rats were run an additional six days of Chain #4 prior to devaluation training. Of this subset, 60% were classified as habitual, whereas only 27% of those trained on the original six days of Chain #4 were classified as habitual. These findings complement those of Zapata et al. (2010) who also showed that extended training on the chained self-administration paradigm led to a greater percentage of rats exhibiting habitual control over their cocaine-seeking. The present results also extend those findings by demonstrating that simply extending the time on the Chain #4 schedule, without taking the animals through extinction and testing first, is a viable approach to promote development of habitual control over cocaine-seeking behavior in this paradigm.

A viral vector under a D2-specific promotor was used to express eYFP for patch-clamp studies, as this has been shown to reliably infect D2 MSNs in ventral striatum (Zalocusky et al., 2016; Soares-Cunha et al., 2022). Importantly, RNAscope/IHC analysis indicated that eYFP expression in our hands using this viral construct was limited to Penk1-expressing MSNs and was not observed to colocalize in Tac1-expressing MSNs, supporting the claim that eYFP+ labeled neurons are D2 MSNs (Fig. 2). As cholinergic interneurons have also been shown to express D2 receptors, it is possible some cells labeled were not MSNs (Kreitzer and Malenka, 2008). However, in all electrophysiological recordings, intrinsic properties of the recorded cells, as well as visual cues, indicated these were MSNs rather than cholinergic interneurons. Specifically, cholinergic interneurons display depolarized resting membrane potentials (−54 to −60 mV) and are clearly identifiable within the slice by their large cell body in comparison with MSNs (Kawaguchi, 1992; Bennett and Wilson, 1999). Further, we did not observe any eYFP+ cells that did not also express Penk1 in our RNAscope/IHC analysis, suggesting eYFP expression is restricted to MSNs, as cholinergic interneurons in the striatum do not express Penk1 (Meredith and Chang, 1994). Together, electrophysiological recordings and RNAscope/IHC analysis suggest the eYFP+ cells in the DLS are D2 MSNs. The specific phenotype of the eYFP− neurons from which we recorded is unknown. However, these cells displayed characteristics consistent with MSNs, indicating that these neurons are likely either D1 MSNs or nontransfected D2 MSNs. Our observations that the RI and PPRs differ between the eYFP+ and eYFP− neurons further suggests that these eYFP− cells are likely a separate population of MSNs (i.e., D1 MSNs).

The present findings suggest excitatory synaptic parameters of the eYFP+ D2 MSNs are unaffected by cocaine self-administration experience whether cocaine-seeking behavior is under goal-directed or habitual control. In the analysis of RI and AMPA:NMDA receptor ratio in eYFP+ cells, there were no significant differences observed between yoked-saline control rats and rats with a history of cocaine self-administration, whether goal-directed or habitual in their cocaine-seeking. This lack of any differences in the RI and AMPA:NMDA ratio indicate that the number and composition of postsynaptic AMPA receptors in D2 MSNs are not changed by cocaine experience (Fig. 4). Similarly, in eYFP− cells, there were no significant differences in RI or AMPA:NMDA receptor ratio, indicating postsynaptic AMPA receptors are also unaltered in this cell population, presumed to be D1 MSNs (Fig. 6).

The lack of changes in excitatory postsynaptic measures in DLS of rats with a history of cocaine self-administration was surprising given that rats clearly differed in the nature of the behavioral control over their cocaine-seeking (Fig. 1). Numerous studies of dorsal striatum have demonstrated synaptic changes associated with the acquisition and expression of goal-directed and habitual behaviors (Amaya and Smith, 2018; Lipton et al., 2019). For example, increases in AMPA:NMDA receptor ratio in D1 MSNs of the DMS is observed in goal-directed behaviors (Shan et al., 2014). However, in habitual behaviors, both D1 and D2 MSNs of the DLS exhibit similar robust synaptic changes (Yin et al., 2009; O’Hare et al., 2016). As such, we expected to observe similar synaptic changes in DLS of our habitual cocaine-seeking rats, yet we did not. Cocaine is known to influence cell-type-specific synapses in a projection-specific manner (Zinsmaier et al., 2022). For example, within the NAc, cocaine self-administration differentially affects synapses from the infralimbic prefrontal cortex (ILC-NAc) and the ventral hippocampus (vHipp-NAc) onto D1 MSNs, with ILC-NAc synapses enriched with CP-AMPA receptors and vHipp-NAc synapses enriched with calcium-impermeable AMPA receptors (Pascoli et al., 2014; Zinsmaier et al., 2022). Therefore, it is conceivable that the lack of differences observed in the present study reflects a similar mechanism of cocaine-induced, afferent-specific changes in the dorsal striatum. Such differences would be obscured in our studies because the local stimulation used did not activate specific afferent pathways. Additional experiments will be needed to identify any pathway-specific changes in excitatory synaptic transmission onto MSNs of the DLS using afferent-selective stimulation approaches, such as with optogenetics. Moreover, as mentioned above, identified D1 MSNs remain to be more thoroughly interrogated, as D1 MSNs in DLS also exhibit activity changes as behavior is learned and expressed, suggesting similar synaptic changes are occurring to D1 MSNs (Smith et al., 2021). It is therefore conceivable that D1 MSN synapses in the DLS may exhibit synaptic strengthening in cocaine-experienced animals and habitual cocaine-seeking behavior.

While the DLS has classically been associated with habitual behaviors, there is a growing body of evidence that indicates the DMS and goal-directed actions are also involved in the development of habitual behaviors (Balleine and O’Doherty, 2010). Recent evidence suggests habits interact with the goal-directed process in a hierarchical manner wherein goal-directed processes select habitual acts to reach the desired outcome, suggesting goal-directed behavior, and thereby the DMS is actively involved in habitual acts (Dezfouli and Balleine, 2012). Moreover, as instrumental behavior is learned and under goal-directed control, D1 MSNs in the DMS exhibit robust synaptic strengthening (Shan et al., 2014; Costa et al., 2017; Peak et al., 2020). In contrast, D2 MSNs of the DMS exhibit synaptic depression when behavior is goal-directed (Shan et al., 2014; Costa et al., 2017). However, as behavior becomes under habitual control, D1 MSNs exhibit synaptic depression (Yu et al., 2021). Together, these observations suggest that both D1 and D2 MSNs of the DMS undergo extensive synaptic changes as behavior is learned and transitions to habitual control. Therefore, both D1 and D2 MSNs of the DMS represent another crucial area of study in the transition from goal-directed to habitual control over cocaine-seeking behavior.

Despite not seeing differences in synaptic strength in eYFP+ D2 MSNs and eYFP-negative MSNs in DLS, we did observe statistically significant differences in short-term plasticity, as measured by PPRs. Specifically, there was weaker paired-pulse facilitation of input to D2 MSNs from rats classified as habitual in their cocaine-seeking relative to rats classified as goal-directed. Further, in the eYFP-negative MSNs, paired-pulse facilitation was stronger in the rats with a history of cocaine self-administration, namely, in the eYFP-negative MSNs from rats characterized as habitual in their cocaine-seeking relative to yoked-saline controls. Paired-pulse facilitation has long been considered a form of short-term synaptic plasticity related to the probability of neurotransmitter vesicle release at the synapse and is, thereby, largely considered to be presynaptic in origin (Cepeda et al., 2008; Glasgow et al., 2019). This classical view of the PPR would suggest, therefore, differential changes in release probability onto D2 MSNs and presumed D1 MSNs in the DLS in rats with habitual control over drug-seeking behavior. However, there are numerous other mechanisms that may contribute to alterations in PPR (Glasgow et al., 2019). For example, some studies have shown that PPR values stay constant as the release probability is altered (Manita et al., 2007; Deutschmann et al., 2022), and seminal work on striatal MSNs has shown dissociation between PPR and other measures of release probability onto D2 MSNs (Cepeda et al., 2008). It is also important to acknowledge that sample sizes from our PPR analyses are small (4–9 cells/group) and the resulting power (0.77 for eYFP+ analyses, 0.73 for eYFP− analyses) slightly below the typical desired power of 0.8. Therefore, the present PPR data suggest the possibility that short-term plasticity in excitatory afferents onto MSNs in DLS may be altered in rats that have developed habitual control over their drug-seeking behavior, and the nature of those alterations may differ between D1 and D2 MSNs. That said, the present data should be considered preliminary, and future work should more thoroughly examine whether differences in short-term plasticity of excitatory input onto D1 and D2 MSNs in DLS are apparent in rats with habitual control over cocaine-seeking behavior.

It is important to note a limitation to the study of RI values in our preparation. Specifically, the preparations in which the RI in eYFP+ and eYFP− cells were determined did not include NMDA receptor antagonists, because we were attempting to maximize data collection from each rat, given the duration of the training required. Consequently, there is the possibility that our RI measures may be confounded by NMDA receptor-mediated currents at depolarized potentials (+40 mV). While AMPA and NMDA receptors display different kinetic properties, with NMDA receptors possessing notably slower rates of synaptic transmission, NMDA receptor-mediated currents at depolarized potentials may still contribute to synaptic responses and, thereby, contaminate the outward AMPA EPSC measured at +40 mV. Importantly, many aspects of NMDA receptors can contribute to rectification patterns, including NMDA receptor subunit composition and extrasynaptic NMDA receptor activation (Sun and Liu, 2007; Jeun et al., 2009). It is possible that NMDA receptors containing the NR2A subunit, which possess faster kinetics than those containing the NR2B subunit, may contribute to the outward EPSC at depolarized potentials (Jeun et al., 2009). To this point, NR2A subunits are expressed at higher levels in DLS of adult rodents and NMDA receptor-mediated currents in the DLS exhibit faster rise times and decay constants relative to ventromedial striatum (Standaert et al., 1994; Chapman et al., 2003). Given this limitation, future studies should assess RI in the presence of APV, an NMDA receptor antagonist, or through the use of the specific CP-AMPA receptor antagonist, Naspm.

This limitation regarding RI notwithstanding, differences in the RI of eYFP+ versus eYFP− neurons were apparent in the yoked-saline controls, with the RI being ∼70% greater in the eYFP− MSNs. Importantly, the RI in the eYFP+ MSNs of the saline controls was ∼1, suggesting no inward rectification and, thus, few CP-AMPA receptors, consistent with expression of the GluR2 subunit in all identified striatal efferent neurons (Deng et al., 2007). The present observation, however, suggests the possibility that eYFP− MSNs—presumed D1 MSNs—in DLS may express more CP-AMPA receptors relative to D2 MSNs (eYFP+ MSNs). While differential changes in CP-AMPA expression in D1 versus D2 MSNs have been reported in the NAc in the context of incubation of cocaine craving (cf. Terrier et al., 2016; Wolf, 2016), to our knowledge no one has heretofore reported differences in RIs in D1 versus D2 MSNs in NAc or dorsal striatum. The present findings therefore raise the intriguing possibility that the eYFP− MSNs (presumed D1 MSNs) in DLS express more CP-AMPA receptors. Of course, the caveat regarding the potential role of NMDA receptor subunit composition still pertains, although to date differences in the expression of NMDA receptor subunits (Standaert et al., 1999; Ganguly and Keefe, 2001) or excitatory synaptic NMDA receptor subtypes between D1 and D2 MSNs have also not been reported (Krajeski et al., 2019). Clearly, additional studies are needed to thoroughly examine AMPA receptor subtype expression and function between these two populations of MSNs in the DLS.

The present study represents the first examination of whether there are MSN-specific adaptations in excitatory synaptic transmission in the DLS of rats classified as goal-directed or habitual in their cocaine-seeking behavior. Overall, we observed that excitatory synaptic strength in D2 MSNs as well as unlabeled, presumed D1 MSNs is not different in rats characterized as habitual versus goal-directed in their cocaine-seeking behavior, either relative to each other or to yoked-saline controls. Therefore, there do not appear to be changes in synaptic strength assessed through AMPA:NMDA receptor ratio or RI in MSNs of the DLS associated with the development of habitual control over drug-seeking behavior. Interesting preliminary data, however, suggest the possibility that changes in short-term plasticity, as evidenced by changes in PPRs, may be associated with the development of habitual control over behavior and that such changes may differ between D2 and D1 MSNs. While cell-type-specific adaptations have been well characterized in the NAc, studies of the cell-type-specific synaptic changes of the dorsal striatum in drug-related behaviors remains in its infancy. Nevertheless, the present study represents a critical starting point for future investigations to the neurobiology of the dorsal striatum in the development and progression of cocaine use behaviors.

## Synthesis

Reviewing Editor: Jean Christophe Poncer, Institut du Cerveau Decisions are customarily a result of the Reviewing Editor and the peer reviewers coming together and discussing their recommendations until a consensus is reached. When revisions are invited, a fact-based synthesis statement explaining their decision and outlining what is needed to prepare a revision will be listed below. The following reviewer(s) agreed to reveal their identity: Nicolas Gervasi, Vincenzo Mastrolia.

1. Most importantly, we feel that the main conclusion of the paper-that there are no significant postsynaptic changes in the DLS between the "goal-directed" and "habitual" groups-is challenged by the design of the experimental protocol. The electrophysiology was performed after a 15+ day devaluation/revaluation period. This long interval and the new behavioral experience may have altered or reversed any plasticity present immediately following the habit-forming training. In addition, the validity of analyzing the rectification index of EPSCs in the absence of pharmacological blockade of NMDARs or AMPAR blockers (e.g., Naspm) is questionned. These limitations call into question the validity of the authors' conclusion and must be addressed experimentally.

2. The main positive effect revealed in this study-a presynaptic change in paired-pulse experiments-appears preliminary, as the sample size for this analysis is too small to be conclusive. Additional recordings are necessary to reach conclusive statistical power and consolidate the conclusion.

3. The method of categorizing animals into "goal-directed" and "habitual" groups-the basis of all comparisons-is questionable, as are the arbitrary 70%/80% cut-offs. The data appear to show a bimodal distribution that differs from the distribution in the cited reference article, suggesting that the cutoff may be incorrect for the author's dataset. A more detailed day-by-day analysis of the data is required, as is a better data-based justification for the cutoff used to distinguish groups based on behavior.

4. The quality of the figures is problematic. In particular, the figures used to validate the identity of dSPNs and iSPNs (e.g., eYFP/RNAScope) are unconvincing. The authors must revise their illustrations and provide files of sufficient quality and resolution.

5. The authors' framing of their work within the existing field is sometimes problematic and biased. References are not always properly cited for their actual conclusions, and reviews are often cited instead of original papers.

## Author Response

Reviewer 1:

1. Throughout the manuscript, the authors repeatedly suggest that synaptic strengthening in iSPNs of the DLS is selectively enhanced relative to dSPNs after extended training, and that this preferential potentiation supports habitual control over behavior. The references cited to justify this claim (Yin et al., 2009; O'Hare et al., 2016) do not support such a conclusion. In fact, both studies report increased synaptic strength in both dSPNs and iSPNs following training. The current interpretation therefore misrepresents these findings and conveys an inaccurate view of the existing literature. This issue appears not only in the introduction but is reiterated in several parts of the manuscript. The authors must correct these statements to accurately reflect the data from the cited studies and avoid implying selective involvement of iSPNs in habitual control without appropriate evidence.

Response: We appreciate the reviewer's comment on inconsistencies in relevant background information. We have made changes to the Introduction and Discussion to accurately reflect the existing literature.

2. The introduction presents goal-directed actions and habitual behaviors as being exclusively mediated by the DMS and DLS, respectively. While this classical framework is well supported by the cited literature, it is not the only model currently discussed in the field. Several studies propose that both DMS and DLS can be engaged during goal- directed and habitual control, with the balance of their involvement depending on task structure, training history, and action-outcome contingencies (e.g., Balleine &O'Doherty, 2010; Dezfouli &Balleine, 2013). I suggest that the authors acknowledge these alternative models to provide a more complete and up-to-date theoretical framework for striatal contributions to action control.

Response: We appreciate the reviewer's comment surrounding alternative models in the field. We have added a paragraph to the Discussion to consider these alternative models.

3. The authors classify animals as goal-directed or habitual based on fixed thresholds of the cocaine-seeking score ({less than or equal to}70% for goal-directed, {greater than or equal to}80% for habitual). Since these thresholds are somewhat arbitrary and task- dependent, I suggest that the authors provide a brief justification for choosing these cut- offs, particularly because they subsequently use this classification for correlations with electrophysiological measures. literature (Zapata et al., 2010; Giangrasso et al., 2023). As these are somewhat arbitrary, we also included correlation data in our figures to show there is no relationship between cocaine-seeking score and electrophysiological measures regardless of the behavioral classification.

4. The manuscript does not provide detailed information on behavioral responses during the 13-day outcome-devaluation period when only the taking lever was available. The authors report only the final lever presses: "All rats reduced responding during devaluation training, with no significant differences observed on the final day of devaluation training between goal-directed (13.1 {plus minus} 2.56 lever presses; n = 17), intermediate (29.3 {plus minus} 9.67 lever presses; n = 3), or habitual (49.5 {plus minus} 28.1 lever presses; n = 12) classifications (Kruskal-Wallis statistic = 4.625; p = 0.099)". The variability in the habitual group appears particularly large compared to goal- directed animals. It would help to confirm that the outcome was effectively devalued if the authors could provide data from the first day of outcome devaluation, showing how lever pressing changed over the course of the devaluation period.

Response: We appreciate this observation and have included taking-lever response data of FR1 training, devaluation, and revaluation as a supplemental figure. We recognize the variability in the habitual group, which is due to one animal on the last day of devaluation only. This animal had 354 lever presses on the final day of devaluation.

5. The authors report that all rats reinstated cocaine-taking behavior during revaluation training (22.8 {plus minus} 1.1 lever presses), similar to initial FR1 training (20.8 {plus minus} 1.37). However, the reported final lever presses at the end of the devaluation period for the habitual group were much higher (49.5 {plus minus} 28.1). It is unclear how the habitual rats' lever pressing decreased so markedly during revaluation compared to the end of devaluation. Providing individual data or separating the revaluation lever presses by classification group would help clarify these apparent discrepancies and ensure consistency between the behavioral classification and the observed lever-pressing behavior.

Response: Please see response above to point 4. days of Chain #4 training to promote habitual responding. It would improve clarity if these animals were explicitly represented in Figure 1, for example as a separate panel (e.g., Panel B), showing their cocaine-seeking scores. This would allow the reader to visualize how extended training affects the distribution of goal-directed, intermediate, and habitual classifications, and how their scores compare to the main cohort.

Response: We appreciate this suggestion and have made changes to Figure 1 to include this distinction.

7. The manuscript reports intrinsic electrophysiological properties for iSPNs (n=28) and dSPNs (n = 21), but it is not specified from which behavioral group these cells were obtained (goal-directed vs. habitual). It would be important to clarify, for each subpopulation, how many cells were recorded from goal-directed versus habitual animals. Additionally, the authors could examine whether intrinsic properties differ depending on the behavioral classification of the animals.

Response: We included all behavioral classifications in these analyses. The manuscript has been updated to include this.

8. The analysis of paired-pulse ratio (PPR) compares n = 9 cells from goal-directed animals with n = 4 cells from habitual animals (without the number of animal for each group). Given these very small and unequal sample sizes, it is difficult to reliably estimate variability or to draw strong conclusions about differences between groups. The authors should acknowledge this limitation and interpret these results with caution.

Response: We acknowledge this limitation of insufficient power and refer to these results throughout the manuscript as preliminary. Unfortunately, the experimenter who performed electrophysiology recordings is no longer with the institution, and the corresponding author's lab has shut down pending retirement. Therefore, additional experiments are not feasible at this time.

9. Since the authors report no detectable synaptic changes in the DLS during the development of habitual drug-seeking behavior, it would be important to discuss alternative loci for plasticity within the dorsal striatum. The absence of plasticity in the DLS does not preclude the possibility that intrinsic or synaptic modifications occur elsewhere in the circuit-particularly in the DMS-that may contribute to the emergence of drug-seeking habits. I recommend including this point in the Discussion to provide a more complete interpretation of the negative findings and to outline potential directions for future investigation.

Response: We recognize the importance of the DMS involved in the expression of goal- directed and habitual behavior and have updated the Discussion to include this point.

10. The authors report no differences in excitatory synaptic strength in the dorsolateral striatum between goal-directed and habitual animals. However, electrophysiological recordings were performed after a long devaluation/re-valuation behavioral protocol (more than 15 days). It is possible that any synaptic modifications that occurred during or immediately after the extended Chain #4 training were altered, reversed, or masked by the devaluation procedure and subsequent testing. The authors should discuss the possibility that the timing of their recordings may not capture the relevant plasticity underlying goal-directed versus habitual cocaine-seeking behavior in the DLS, and how this might influence interpretation of their negative findings.

Response: We appreciate timing may have a significant impact on the findings reported and that it is possible synaptic modifications may occur that are masked by devaluation training. However, due to the nature of the paradigm, there is no way to determine behavioral classifications prior to outcome devaluation. As the primary objective of the study was to assess synaptic changes associated with habitual cocaine-seeking behavior, the devaluation procedure is necessary to categorize behavioral control over cocaine- seeking. In our consideration, given the paradigm, there is no way to address this possibility.

11. The quality of the figure showing eYFP expression and RNAScope/IHC labeling is too low to confidently support the claim of selective expression in iSPNs.

Response: High resolution images have been provided for all figures.

12. In all the manuscript the authors should report both the number of cells and the number of animals from which these cells were obtained for all electrophysiological recording to ensure transparency and allow proper assessment of statistical validity (i.e n=28 from 10 animals).

Response: We have included these values in each of the figures and figure legends.

13. The Materials &Methods section should include the titer of the virus and the exact volume injected, in addition to the virus ID, to ensure reproducibility of the viral injections.

Response: The exact volume injected and virus titer have been included in the Methods section.

Reviewer 2:

1. All figures, including their legends, are currently in low resolution, which makes it difficult to evaluate the data accurately. Please update all figures to a higher resolution (typically 300 dpi) to facilitate proper assessment.

Response: High resolution images have been provided for all figures.

2. Habituation vs goal-directed definition. The manuscript follows the classification present in Giangrasso et al. 2023 to define the different cocaine-seeking behavioural categories, with the cocaine-seeking score cut-off at 70% used to distinguish between goal-directed and habitual. This classification also includes an intermediate category, which is however not considered further in the study for lack of number of animals. I recommend incorporating the animals classified as intermediate into one of the main categories to strengthen the dataset. Furthermore, by looking at Giangrasso et al 2023, the distribution of their cocaine-seeking scores looks unimodal, with the 70% cut-off cutting through the middle of the distribution. The current manuscript, however, shows a clear bimodal distribution which would suggest a different cocaine-seeking score at a different value of 100% circa. I suggest performing additional behavioural tests to confirm whether this bimodal distribution persists in your data. If so, adjusting the cut-off accordingly may strengthen your results.

Response: Unfortunately, the lab in which experiments were performed has shut down, preventing the possibility of additional studies. We did have a small number of animals that were classified as "intermediate" based on their cocaine-seeking scores. However, after data exclusions, not enough animals were classified as intermediate to be included in analyses.

3. Please provide additional details in the figures related to the behavioral tests (figure 1), including more comprehensive plots. In addition, some schematics would be very useful to the readers to grasp the behavioral paradigms protocols.

Response: We have improved the quality of Figure 1 to include a distinction for the subset of animals trained for an additional six days on Chain #4.

4. The study currently includes only male rats. Including a small cohort of female rats would strengthen the findings by confirming their applicability across sexes and align the study with current best practices regarding sexual dimorphism. While not a strict requirement for acceptance, this addition would enhance the manuscript.

Response: As mentioned above, the lab in which all behavioral experiments were performed has shut down due to pending retirement of the PI. Therefore, despite the need for the study of female rats in this context, additional studies are not possible at this time.

5. In figure 2, the DAPI staining used seems very odd. It is extremely difficult to distinguish the nuclei from the rest of the cytoplasm. For example, in fig 2A eYFP fully colocalizes with DAPI staining, with no visibility for any cytoplasmic area. Furthermore, fig 2B shows a Tac1-negative, YFP-positive cell with a barely visible nucleus. The authors should spend more time in expanding and refining the images used in figure 2, as it is a critical result used to define the identity of D2-MSNs.

Response: A high resolution image of figure 2 has been provided.

6. Figures 3 to 6 would benefit from additional analysis of evoked EPSCs, particularly regarding current kinetics. This may reveal further differences in kinetics-related parameters.

Response: We appreciate that kinetic analyses of evoked EPSCs may reveal differences otherwise not apparent. Kinetics-related parameters including rise-time, for example, would provide insight to neurotransmitter release and may further reflect AMPA and/or NMDA receptor subunit changes. However, a previous study referenced in the manuscript (Giangrasso et al., 2023) determined no changes in indices of presynaptic glutamate release in both goal-directed and habitually classified animals in relation to saline controls. Moreover, as pharmacology was lacking from our EPSC recordings, differences in kinetic analyses would need to be further confirmed with specific AMPA and NMDA receptor antagonists. As mentioned, we are unable to perform additional experiments to include these pharmacological assessments.

7. I commend the authors' honesty in the discussion, especially in discussing the weaknesses pf the RI analysis. Upon their admission, the authors did not use specific blockers to isolate the CP-AMPA mediated currents. The justification for this is unfortunately weak: "the preparations in which the RI in eYFP+ and eYFP- cells were determined did not include NMDA-receptor antagonists, because we were attempting to maximize data collection from each rat, given the duration of the training required." My guess would be that looking at the RI was an afterthought when the experiments were already completed. If the authors would have considered this aspect during the experiments, it would have been very easy to combine the pharmacology with the other electrophysiological experiments, adding APV or Naspm as the final step in each experiment. Therefore, at least as a small testing group, I would invite the authors to perform a few recordings in the presence of these blockers, to solidify the conclusions of the study about CP-AMPA, which are for the moment only weak suggestions.

Response: Initial experiments explored the inclusion of pharmacological manipulations; however, prolonged drug perfusion led to cell loss over the course of the recordings. Given the fragility of cells within brain slices prepared from older animals, and the considerable time and effort required for animal training, we prioritized maximizing data acquisition under stable recording conditions and therefore proceeded without pharmacology experiments. Notably, analyses of RI revealed no significant differences across conditions. Had such differences emerged, pharmacological experiments would have been critical to the interpretation of those findings.

8. The results in figure 7 on presynaptic with PPR analysis are the only positive results of the manuscript. The sample size for eYFP+ cells in the habitual behavioural category is low. Increasing the number of recordings in this group would help solidify the conclusions.

Response: Please see response to Query 8 of reviewer 1 above. Also as previously noted, additional experiments are not possible due to closure of the lab in which the behavioral work was done and the move of the individual who performed the electrophysiological recordings from the university.

Minor comments 1. In the introduction, second paragraph and important point for the paper, about changes in GABAergic synapses in the DLS cites only 2 reviews. The authors should directly cite the research papers and not just the reviews. This is an important issue across the whole introduction, with a considerable number of reviews used for citations.

Response: We have adjusted citations and references to include primary research papers.

2. Please consider adding a supplementary table listing all behavioural results parameters, not just the statistical tests used.

Response: We have included a supplemental figure with all taking lever responses that includes all phases of the behavioral paradigm.
