# Supplementary figures and images for: Assessment of Cell-Type-Specific Excitatory Synaptic Strength in the Dorsolateral Striatum of Goal-Directed and Habitual Cocaine-Seeking Behavior
Source: eNeuro. 2026 May 22;13(5):ENEURO.0392-25.2026. doi: 10.1523/ENEURO.0392-25.2026 (PMC13211988; doi:10.1523/ENEURO.0392-25.2026)

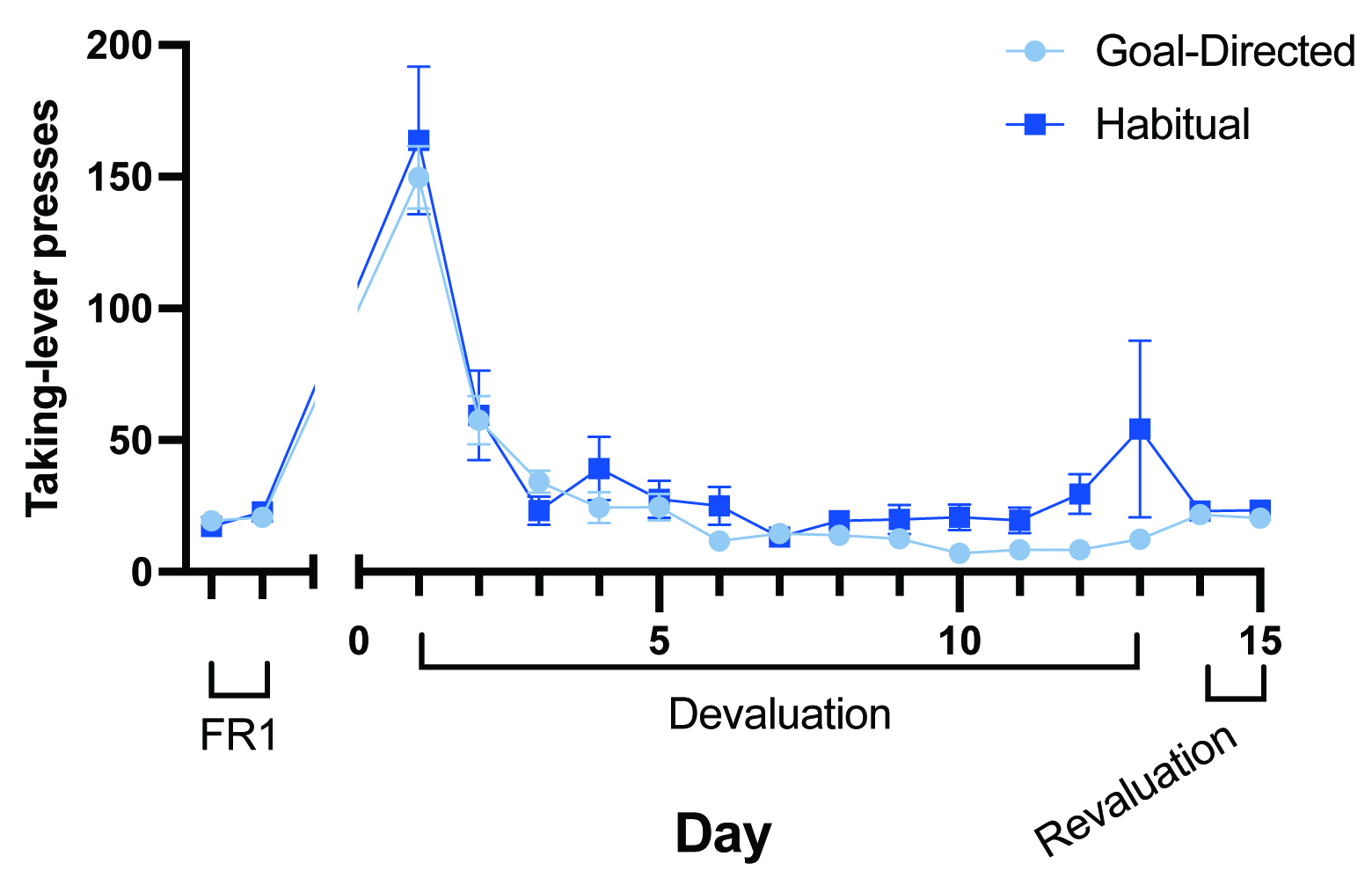

Supplement: Figure 1-1 — Taking-lever presses throughout cocaine self-administration training, devaluation (i.e., extinction), and revaluation sessions in rats ultimately classified as goal-directed (n = 11) or habitual (n = 10) in their cocaine-seeking behavior. All sessions were two-hours in length, with the exception that responding in the FR1 and Revaluation sessions was restricted to a maximum of 40 lever presses and, thus, could have been terminated at less than two hours. Data are represented as mean + SEM. Download Figure 1-1, TIF file. [file eneuro-13-ENEURO.0392-25.2026-s002.tif]

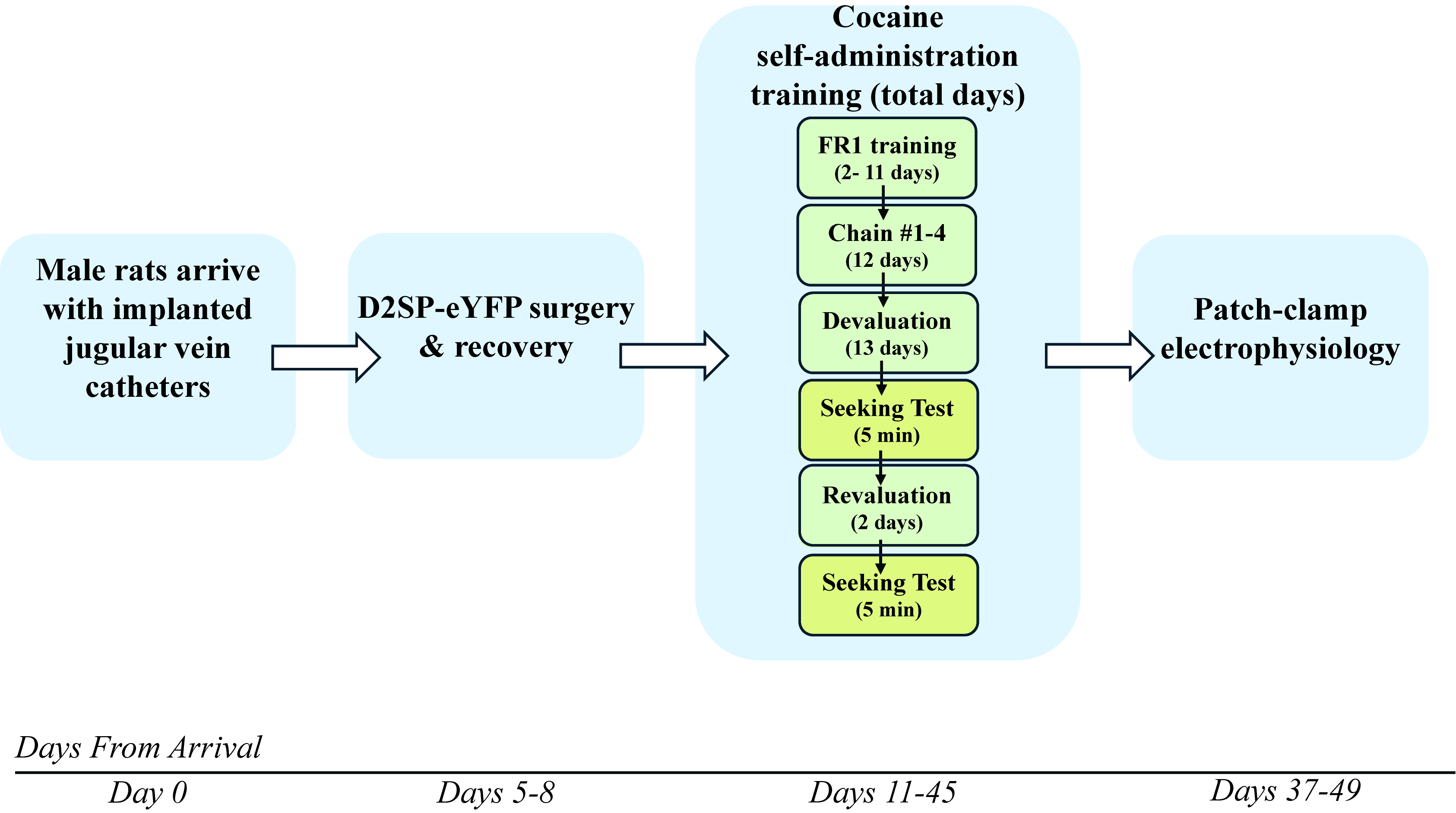

Supplement: Figure 1-2 — Schematic of experimental paradigm. Rats arrived implanted with jugular vein catheters and underwent surgery within 3-5 days of arrival. Following recovery, rats then advanced to cocaine self-administration training. FR1 training duration was dependent upon meeting criteria of two consecutive sessions of >10 infusions. Within 4 days of the final testing session, rats were used in electrophysiology experiments. Download Figure 1-2, TIF file. [file eneuro-13-ENEURO.0392-25.2026-s003.tif]
